# Discovery of Clonixeril as a Sub-Femtomolar Modulator of the Human STING Receptor

**DOI:** 10.1021/acscentsci.4c01982

**Published:** 2025-06-06

**Authors:** Robert P. Sparks, William Lawless, Anna Kharitonova, Rainer Metcalf, Jamie Nunziata, Grace A. Binder, Sauradip Chaudhuri, Christine S.R. Gambino, Michelle Wilde, Linette S. Harding, Jaret J. Crews, Mansi Gopu, Emilia Dalamangas, Sarah Lawless, Mark Eschenfelder, Robert M. Green, Elizabeth X Nompleggi, Timothy H. Tran, Yan Yang, Donna V. Trask, Paul R. Thompson, Rekha Patel, Niketa A. Patel, Wesley H. Brooks, Guy Bradley, Mildred E. Acevedo-Duncan, Alan C. Mullen, James W. Leahy, Kenyon G. Daniel, Wayne C. Guida

**Affiliations:** † Department of Chemistry, 7831 University of South Florida, Tampa, Florida 33620, United States; ‡ Research Service, 19995 James A. Haley Veterans Hospital, Tampa, Florida 33612, United States; § Division of Gastroenterology, Department of Medicine, 12262 University of Massachusetts Chan Medical School, Worcester, Massachusetts 01655, United States; ⊥ Department of Molecular Medicine, Morsani College of Medicine, 7831 University of South Florida, Tampa, Florida 33620, United States; ¶ Biochemistry and Molecular Biotechnology Department, 12262 University of Massachusetts Chan Medical School, Worcester, Massachusetts 01655, United States; ∥ H. Lee Moffitt Cancer Center, Research Institute at the University of South Florida, Tampa, Florida 33612, United States; # College of the Holy Cross, Worcester, Massachusetts 01610, United States; & Tampa Bay Research Institute, St. Petersburg, Florida 33716, United States; ◆ Florida Center for Drug Discovery and Innovation, 7831 University of South Florida, Tampa, Florida 33620, United States; ○ Department of Molecular Biosciences, 7831 University of South Florida, Tampa, Florida 33620, United States

## Abstract

Stimulator of interferon genes (STING) is a transmembrane endoplasmic reticulum (ER) resident protein involved in innate immunity. STING activation occurs by binding of cyclic guanosine-(2′→5′)-monophosphate-adenosine-(3′→5′)-monophosphate (2′,3′-cGAMP) to STING, which leads to downstream production of type 1 interferons (IFN-1). We generated molecular dynamics (MD) equilibrated agonist and antagonist models of human STING (hSTING) for computer-based screening and now report the discovery of clonixeril (CXL) as the most potent non-nucleotide hSTING modulator discovered to date. We demonstrate *in vitro* and *in cellulo* that CXL has two modes of interaction with hSTING, one with an EC_50_ above 1 nM and the other with an EC_50_ in the 1 fM–100 aM range (10^–15^–10^–16^ M). In cell-based experiments, when CXL is titrated below 1 nM, it displays inverse dose-dependent antagonistic behavior toward hSTING. We have substantiated that CXL displays this exceptionally strong inhibitory effect on hSTING mediated IFN-1 production using a THP-1 cell luciferase reporter for interferon regulatory factor 3 (IRF3). Further characterization of CXL was performed in HEK293 cells by using biophysical and biochemical techniques.

## Graphical abstract

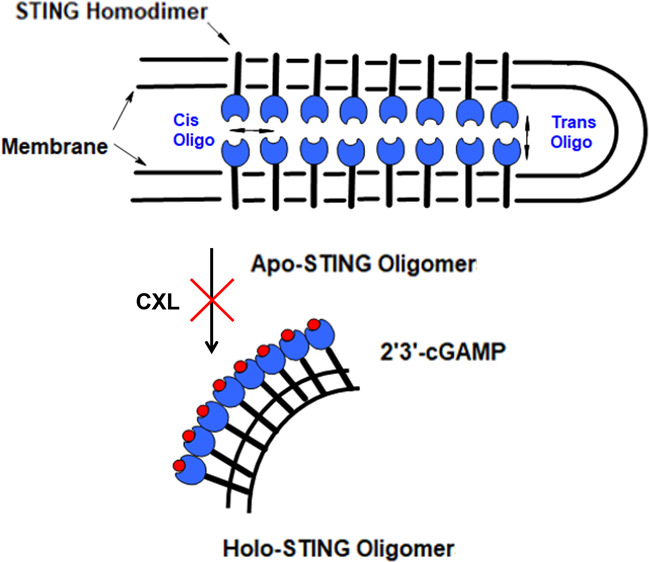

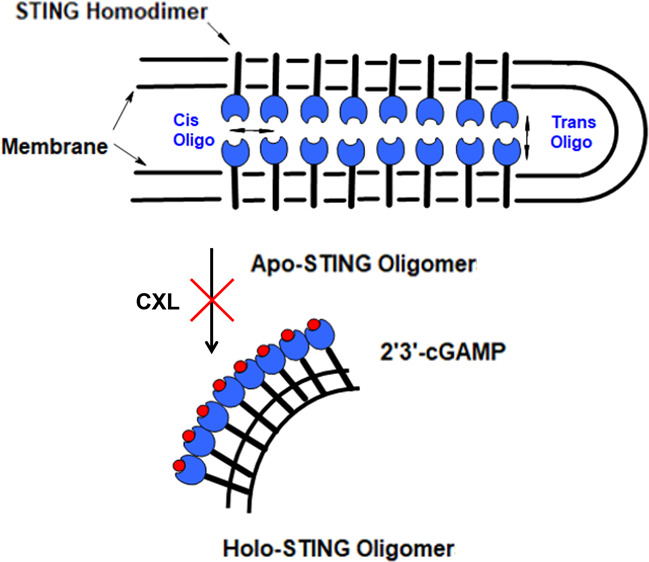

## Introduction

In mammalian cells, recognition of cytosolic DNA occurs largely through the cyclic GMP-AMP synthase enzyme (cGAS), which functions upstream of the stimulator of interferon genes (STING) protein.^1^ Activation of the cGAS-STING pathway results in activation of the innate arm of the immune system.^1^ It is cGAS, a surveillance protein, that produces cyclic GMP-AMP (cyclic guanosine-(2′→5′)-monophosphate-adenosine-(3′→5′)-monophosphate or 2′,3′-cGAMP) as the endogenous activator of the STING pathway (see Figure 1A for chemical structures).^1–3^ cGAS is widely distributed throughout subcellular sites, including the cytosol, the inner leaflet of the plasma membrane, and the nucleus.^2,3^ There are a substantial number of DNA sources that can trigger cGAS enzymatic activity such as from bacteria or a virus.^4^ Moreover, mitochondrial dysfunction, augmented rates of cellular apoptosis, and disturbance of phagocytic digestion in combination with DNase deficiencies, like TREX1 mutations can result in cGAS activation.^4^

**Figure 1:**
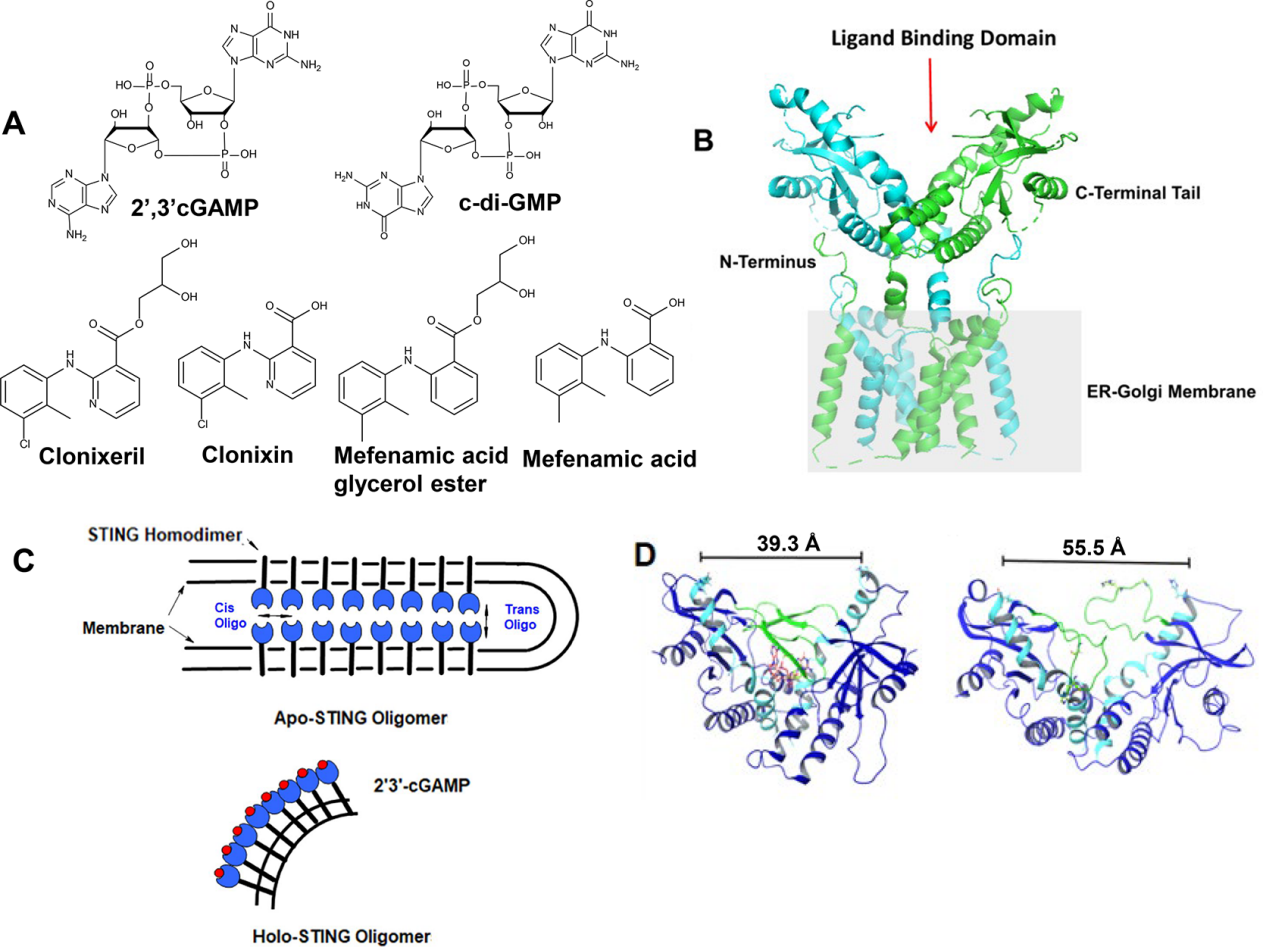
Chemical and protein structures. (A) Chemical structures of known STING nucleotide activators plus clonixeril (CXL), clonixin (CXN) and their analogues, mefenamic acid (MFA), and mefenamic acid glycerol ester (MFE). (B) Cryo-EM structure of full length apo-hSTING (R232). PDB: 6NT5 at a resolution of 4.1 Å. Illustrates the protein ribbon structure with annotations of specific structural components (Left). Overlay the STING protein surface onto the STING ribbon structure (Right). The gray box represents the ER membrane.^5^ (C) Model of STING oligomerization. Depicts two known forms of STING oligomerization, either *cis* or *trans*; generalized after Liu, et al.^6^ Apo-STING and Holo-STING are depicted. (D) Distances of the across nucleotide binding region of STING taken from PDB: 4KSY minimized using Schrödinger software and subjected to MD simulations which indicate a closed (Left) 2′,3′-cGAMP bound state (red carbons) and an open apo state (Right) of STING. “Lid” region demarcated with green ribbon and α1 and α2 helix in light blue.^7^

STING is a mediator of innate immunity, including induction of pro-inflammatory cytokine expression, autophagy, and lysosomal cell death.^8,9^ STING is an endoplasmic reticulum (ER) resident protein that is, in its resting state, locally retained within the ER by a Ca^2+^ sensor, stromal interaction molecule 1 (STIM1).^10,11^ Note, though, that this has been challenged on the basis of recent CryoEM studies.^5^ STING possesses a transmembrane domain that spans the ER membrane (Figure 1B).^5,11^ The carboxy terminal domain (CTD) bears two major amino acid motifs, a highly conserved PLPLRT/SD motif for binding of TANK-binding kinase 1 (TBK1), along with an adjacent pLxIS motif, essential for recruitment and phosphorylation of interferon regulatory factor 3 (IRF3).^12–15^ Under steady state conditions, STING forms a domain-swapped homodimer so that its ligand binding domains (LBD’s) create a V-shaped site suitable for binding of one cyclic dinucleotide (CDN).^7,16^ In the apo state, STING is oligomerized within the folds of the ER in a head to head manner (referred to as *trans* oligomers) and lateral side to side (*cis*) oligomers (Figure 1C).^6^ This zipper-like geometry maintains STING in an autoinhibitory state and ER resident state until activated by 2′,3′-cGAMP.^6^ Binding of 2′,3′-cGAMP induces formation of fully closed dimer angles and the LBD’s rotation in relation to the transmembrane domain, bringing about a shift in geometry that permits the STING bilayer to split into bent active STING monolayers to provide highly condensed *cis* oligomers consisting of lateral stacking of the now fully closed STING dimers.^6,7^

The bent holo structure shown in Figure 1C, ultimately forms a vesicle that is transported to the Golgi.^6^ Additionally, formation of the protein–ligand complex induces a conformational change involving repositioning of the STING C-terminal tail (CTT).^6^ The CTT provides a second autoinhibitory mechanism which results in the protection of the cis oligomer interface, thereby discouraging condensed *cis* oligomerization from prematurely taking place.^6^ The condensed *cis* oligomeric structures are stabilized *via* disulfide linkages between vicinal C148 units, which is imperative for STING to gain signaling competence.^6,17^ These oligomers properly position the kinase domains of one TBK1 dimer relative to another TBK1 dimer (located on an adjacent STING dimers) so that trans-phosphorylation of TBK1 can occur.^18^ Subsequent phosphorylation occurs when TBK1 exerts its catalytic activity on the S366 residue within the pLxIS motif of the neighboring STING dimer.^13^ The endoplasmic-reticulum-Golgi intermediate compartment (ERGIC) serves as an origination site for IRF3 activation.^19^ Phosphorylation of STING’s pLxIS motif initiates recruitment and binding of IRF3 molecules, positioning them near the active site of TBK1 where phosphorylation of IRF3 occurs.^5,13,15^ As STING travels through the compartment, the greatest signal transduction is achieved by means of further cluster formation upon arrival of STING at the Golgi.^20^ Once phosphorylated, IRF3 undergoes dimerization and subsequent nuclear translocation where it binds to promoter regions to induce type 1 interferon (IFN-1) production.^15^ Downregulation of aberrant cGAS-STING activity could alleviate a wide range of inflammatory disorders including the lethal complications that arise from the cytokine storm that can follow SARS-CoV-2 infections.^21–25^

Here, we describe the discovery of clonixeril (CXL) as a potent inhibitor of hSTING, with activity even at attomolar (10^–18^ M) concentrations. Cellular assays using THP-1 and HEK293 cells were performed to assess the effect of CXL on hSTING *in cellulo*. Further characterization of CXL was performed using surface plasmon resonance (SPR), microscale thermophoresis (MST), dynamic light scattering (DLS), and isothermal titration calorimetry (ITC) and native PAGE to study the interaction between CXL and hSTING.

## Results

To identify a small molecule that was not a cyclic dinucleotide but could modulate STING activity, we employed computational modeling commencing with molecular dynamics (MD) equilibrated crystal structures for the C-terminal domain (CTD) of human wild-type STING (hSTING^WT^ CTD, Figure 1D). Computational models developed for hSTING^WT^ agonists and antagonists informed selection of candidates that were initially screened using a differential scanning fluorescence (thermal shift) assay, followed by a THP-1 cell luciferase reporter assay for the pIRF3 dimer binding to its promoter. We identified a low molecular weight compound from the NCI Diversity Set (available from the National Cancer Institute), NSC 335504 (clonixeril, abbrv. CXL), as a potential modulator of hSTING^WT^. We determined that CXL has weak agonist activity at micromolar concentrations and further found that administration of CXL at low femtomolar (10^–15^ M), and in some cases even attomolar (10^–18^ M) concentrations, resulted in antagonism of the hSTING^WT^ pathway. Notably, clonixin (CXN; Figure 1A), which is the carboxylic acid precursor of CXL, exhibited no antagonistic effect nor did it exhibit appreciable agonist activity in our luciferase reporter assay. Our data suggest CXL to be the most potent hSTING^WT^ antagonist reported to date with unprecedented potency in the high attomolar range.

### Computational Model Construction

In general, wild-type hSTING, C-terminal domain (CTD) structures were employed for computational and biophysical studies (subsequently, we refer to this as the hSTING CTD). We employed MD simulations to better understand how the hSTING CTD interacts with endogenous ligands and other potential binding partners using PDB: 4EMU apo-hSTING and PDB: 4KSY; 4F5Y holo-hSTING CTD structures. The distance between the α-carbons of H185A and H185B residues at the end of the α2 helices in dimeric hSTING CTD was used as a metric for full-length hSTING activation. Crystal structures of known agonists exhibited α-carbon distances in the range of 34 to 38 Å for holo structures, whereas apo crystal structures had α-carbon distances in the range 47 to 56 Å. This prompted the generation of two separate docking models (Figure 1D) to screen for (a) agonists, compounds that have greater affinity for the holo (i.e., 2′,3′-cGAMP bound structure, PDB: 4KSY) and (b) antagonists, compounds with greater affinity for the apo structure (i.e., c-di-GMP, PDB: 4F5Y). Current docking programs are restricted to docking and evaluating single molecules at a time. As a result, docking algorithms are unable to effectively handle ligands that bind as dimers, such as the binding of DMXAA to mouse STING.^26^ To overcome this limitation, we developed a simple docking method that can be used in conjunction with standard virtual screening protocols to assist in identifying potential small molecule dimer-protein complexes (see ).

### Computational Docking Using hSTING Models

Docking was performed on both MD equilibrated hSTING CTD antagonist and hSTING CTD agonist models (Figure 1D). Virtual screening of the NCI Diversity Set was performed using the GLIDE docking program (Schrödinger, Inc.). Also, a ligand-based algorithm (Pharmer) was employed for pharmacophore guided virtual screening of larger compound libraries.^27^ The pharmacophore was derived from the X-ray structure of hSTING CTD with 2′3′-cGAMP bound (PDB:4LOH), and the entire ZINC database was screened.^28^ Then, ∼4000 pharmacophore-matched molecules were docked to our hSTING CTD computer models. Next, we examined the top ranking “hits” from virtual screening and subsequently the pharmacophore-based hits *via* a thermal shift assay (), which led to the discovery of CXL as a possible hSTING binder. The site-restriction docking protocol we developed () suggests that two CXL molecules bind to the hSTING CTD ().

### Clonixeril Demonstrates Unprecedented Potency in the THP-1 Luciferase Reporter Assay

Initially we were encouraged because CXL exhibited agonist activity at micromolar concentrations in THP-1 cells (Figure 2A) and, thus, could potentially serve as a lead compound to develop more potent agonists. However, upon further experimentation, we were surprised to discover that decreasing CXL to femtomolar concentrations resulted in antagonism of the hSTING pathway (Figure 2B). Accordingly, the WT-THP-1 QUANTI-Luc luminescence IRF3 reporter assay was employed in which THP-1 cells were treated with CXL at concentrations ranging from 1 nM–100 aM for one hour followed by treatment with 4 μM 2′,3′-cGAMP for an additional 19 h. The results are shown in (Figure 2B), and demonstrate an inverse dose response where lower concentrations of CXL are associated with a decreased response of hSTING to 2′,3′-cGAMP activation.

**Figure 2:**
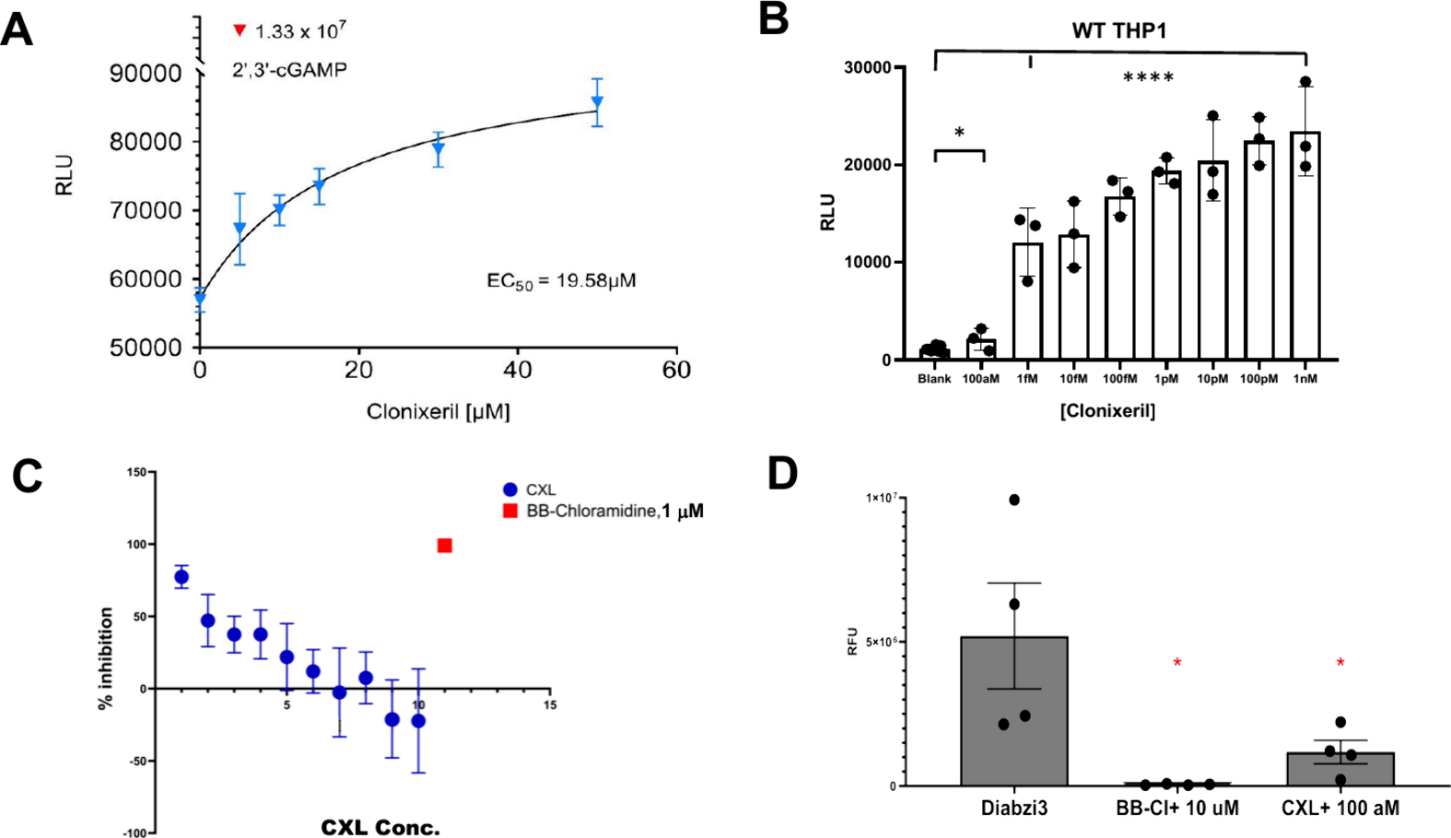
Luciferase assay utilizing monocytic leukemia (THP-1) cells. Cells (Invivogen THP1 Dual KI-hSTING-R232; wild type) were analyzed for activation of the hSTING WT pathway *via* an IRF3 luciferase reporter. Luminescence is reported in relative luminescence units (RLU). Error bars are SEM A. Dose–response of THP-1 cells treated with various concentrations of clonixeril (CXL); *N* = 6. The red data point indicates the response of 2′,3′-cGAMP positive control at 50 μM. B. Competition of THP-1 cells treated first with CXL (1 h) and subsequently treated with 2′,3′-cGAMP 4 μM (9 h); *N* = 3. C. Dose–response of THP-1 cells treated with CXL or BB-chloramidine, a known STING antagonist, in the presence of 50 nM diABZI3. Dose range was from 100 aM to 100 nM (1 = 100 aM, 2 = 1 fM, 3 = 10 fM, 4 = 100 fM, 5 = 1 pM, 6 = 10 pM, 7 = 100 pM, 8 = 1 nM, 9 = 10 nM, 10 = 100 nM; *N* = 4. Results were plotted in GraphPad Prism. D. Quantification of Figure 2C with 100 aM clonixeril and 10 μM BB-chloramidine in the presence of 50 nM diABZI3. *p-value < 0.05 via one way ANOVA test generated from GraphPad Prism.

To validate our initial observations that CXL has extraordinary potency for a small molecule antagonist, we extended our THP-1 cell luciferase reporter experiment by using diABZI3 as an agonist and using BBCl-amidine (a previously reported STING antagonist) as an antagonist control.^23,29^ Furthermore, because the results were so unprecedented, we conducted these experiments in a different laboratory (U. Mass Chan. vs Univ. So. FL) and with different individuals performing the experiments. DiABZI3 was used as a STING activator because it is highly potent, it is cell membrane permeable, and it provides an alternative activator to 2,′3′-cGAMP, which is not stable to phosphodiesterases.^23,30^ Thus, Invitrogen’s WT-THP-1 QUANTI-Luc luminescence assay was performed using 50 nM diABZI3 in the presence of CXL at concentrations ranging from 100 nM to 100 aM. These results confirmed the extraordinary potency of CXL, and the inverse dose response previously observed (Figure 2 B). It is noteworthy that as shown in Figure 2C, higher concentrations of CXL result in enhancement of the diABZI3 signal.

### Clonixeril Inhibits 2′,3′-cGAMP-Dependent Production of p-hSTING in HEK293 Cells

Since phosphorylation of Ser366 of hSTING is a necessary step to initiate recruitment, subsequent docking, and phosphorylation of IRF3, we investigated whether CXL would affect hSTING phosphorylation levels *in cellulo*. Thus, HEK293 cells were treated with varying concentrations of CXL (1fM-1nM) for one hour prior to 2 μM 2′,3′-cGAMP treatment for 90 min (Figure 3A) or 2 h (Figure 3C). An optimized dose of 2 μM 2′,3′-cGAMP was based on a dose–response study done for this experiment. A control group was treated with vehicle for 1 h followed by 2 μM 2′,3′-cGAMP for 1 or 2 h (Figure 3C) Total protein lysates were collected and analyzed by Western blotting using antibodies against p-hSTING, hSTING, and β-actin. The Western blot data demonstrated a drastic increase in hSTING phosphorylation levels in the positive control groups. Treatment with varying concentrations of CXL prior to 2′,3′-cGAMP downregulated hSTING phosphorylation close to near basal cellular levels (Figure 3A–C). Notably, when HEK293 cells were treated with 2′,3′-cGAMP prior to CXL, inhibitory activity was not observed in the same concentration range (Figure 3B).

**Figure 3:**
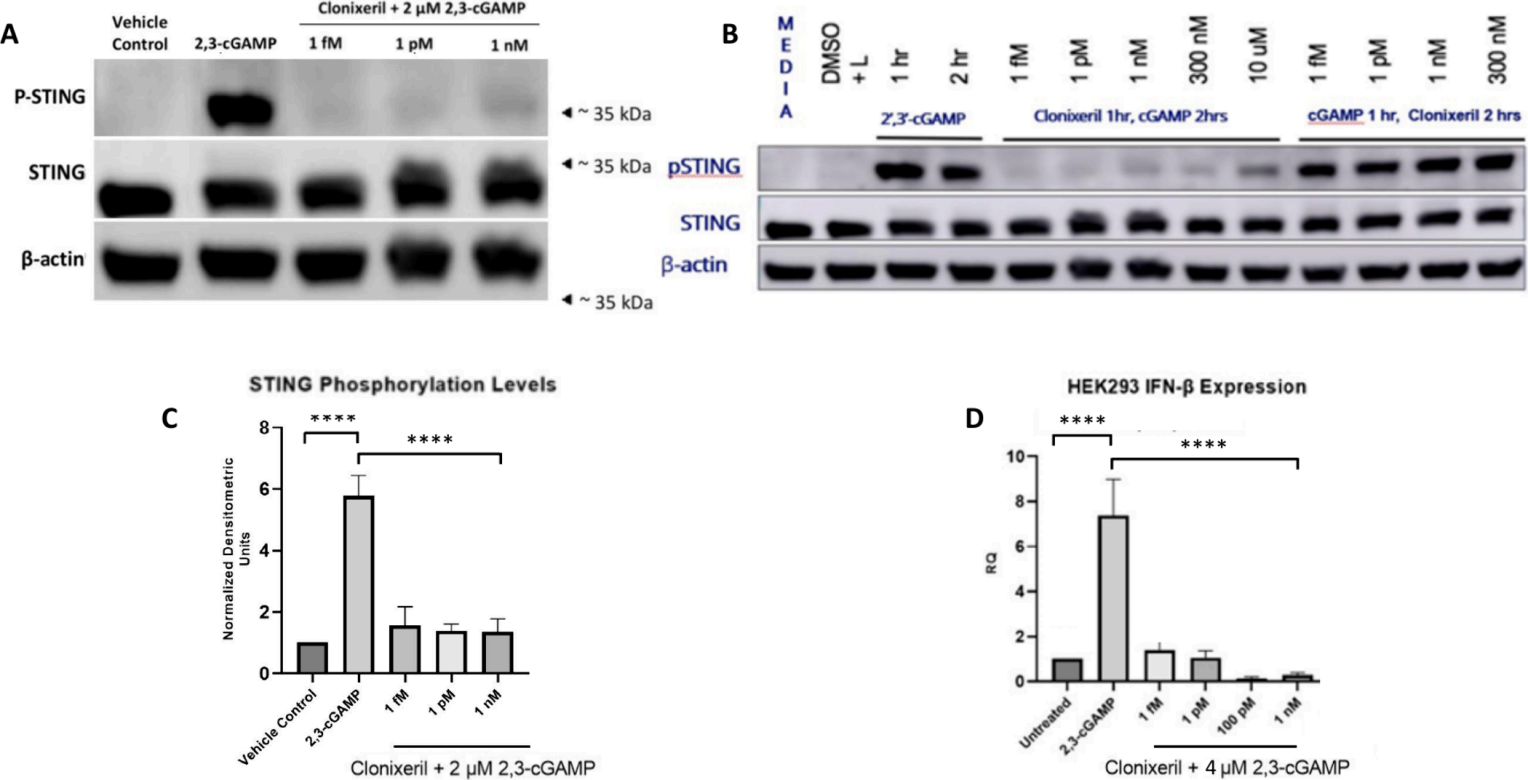
Effects of Clonixeril on STING phosphorylation and IFN-β production. Western Blot analysis for STING and pSTING, qPCR of HEK293 treated with 2′,3′-cGAMP and clonixeril, and clear native PAGE for STING. Data points were analyzed with a one-way ANOVA test using GraphPad Prism. (A) Western blot of HEK293 cells treated with various concentrations of clonixeril followed by treatment with 2 μM 2′,3′-cGAMP; *N* = 3. (B) Western blot of HEK293 cells treated with various concentrations of clonixeril followed by treatment with 2 μM 2′,3′-cGAMP; *N* = 3. (C) Quantitative analysis of Figure 3A. (D) qPCR data for IFNβ levels in HEK293 cells treated with various concentrations of clonixeril followed by treatment with 4 μM 2′,3′-cGAMP; *N* = 5.

### Clonixeril Inhibits 2′,3′-cGAMP-Dependent Production of IFN-β in HEK293 Cells

Various genes comprise the IFN-1 family, including IFN-α, IFN-β, IFN-ω, -ε, and -κ.^31,32^ IRF3 activation leads to the induction of a strong IFN-β response. Thus, we proceeded to evaluate whether CXL would affect the IFN-β levels. HEK293 cells were treated with CXL (1 fM-1 nM) for 1 h followed by 4 μM 2′,3′-cGAMP for 3 h. Cells treated with 4 μM 2′,3′-cGAMP were used as a positive control. Four μM 2′,3′-cGAMP was an optimized dose based on a dose–response study for this experiment. IFN-β expression levels were measured using real-time qPCR. We observed a 7.4-fold induction of IFN-β production in the positive control group. All CXL treatment groups diminished 2′,3′-cGAMP-induced expression of IFN-β by more than 50% including a concentration of 1 fM (Figure 3D).

### Fluorescence Microscopy Experiments Suggest that CXL Affects hSTING Oligomerization

STING undergoes condensed *cis*-oligomerization as part of its signal transduction pathway and punctate structure formation of p-hSTING in the perinuclear region is indicative of hSTING oligomerization.^12^ We had speculated that CXL might affect hSTING oligomerization. Hence, in a preliminary study, we employed immunofluorescence to visualize how distribution of p-hSTING would be affected by treatment with CXL. HEK293 cells were treated with either 10 aM or 1 pM CXL for 1 h followed by 2 μM 2′,3′-cGAMP for 90 min. Cells treated with vehicle control for 1 h and then 2 μM of 2′,3′-cGAMP for 90 min were used as a positive control. At a 1 pM concentration of CXL, both p-hSTING and punctate structure formation were diminished. In addition, our results suggest that CXL treatment at a concentration of 10 aM may affect 2′,3′-cGAMP induced p-hSTING puncta formation in the perinuclear region () but we cannot definitively claim that this is the case based upon these results. However, levels of hSTING phosphorylation were qualitatively unaffected by 10 aM CXL treatment.

### In vitro Characterization of 2′3′-cGAMP’s Effect on hSTING CTD Oligomerization Using Biophysical Methods

#### Dynamic Light Scattering (DLS) and Mass Photometry

Based upon our fluorescence microscopy studies (), we hypothesized that CXL may affect hSTING oligomerization. DLS was used to determine how CXL affects our His-SUMO-TEV-hSTING CTD construct (subsequently referred to as the SUMO-hSTING CTD). These STING oligomerization experiments were conducted under conditions in which 2′,3′-cGAMP is present, as is the case for our competition experiments. The data presented in Figure 4A suggest that CXL alters the ability of SUMO-hSTING CTD to oligomerize in the presence of 2′,3′-cGAMP. It is noteworthy that this effect is observed with concentrations of CXL as low as 100 aM. Interestingly, the interaction of CXL alone with SUMO-hSTING CTD causes a moderate amount of oligomerization within the same concentration range (Figure 4B). This observation demonstrates that CXL has a propensity to cause oligomerization on its own. Importantly, neither H-151, a known STING covalent antagonist that functions upstream of the CTD, nor CXN showed reduction in 2′,3′-cGAMP driven SUMO-hSTING CTD oligomerization or an increase in SUMO-hSTING CTD oligomerization without 2′,3′-cGAMP present (Figure 4A,B). To support the premise that we were measuring STING CTD oligomers *via* DLS, we employed mass photometry, a different method for the detection of protein oligomerization. Accordingly, SUMO-hSTING CTD alone and SUMO-hSTING CTD with 2′,3′-cGAMP (at the same concentrations as used for our DLS experiments) showed results entirely consistent with our DLS studies.

**Figure 4:**
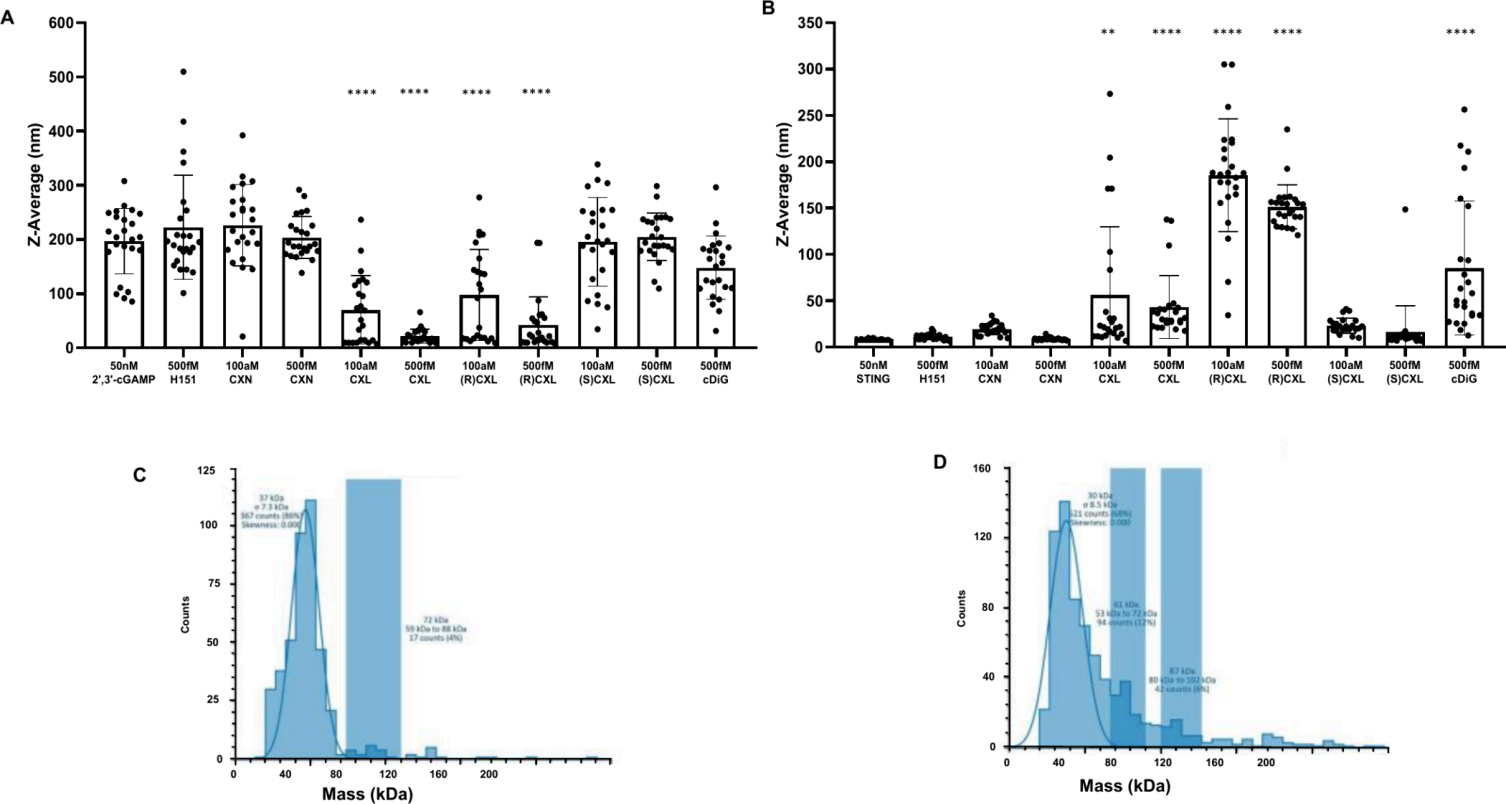
Dynamic light scattering and mass photometry measurements of STING oligomerization. (A) DLS was performed for 2 h (*n* = 960 measurements) with 50 nM STING and 50 nM 2′,3′-cGAMP exposed to analytes. (B) DLS was performed for 2 h (*n* = 960 measurements) with 50 nM STING exposed to analytes. (C) Mass photometry of STING without 2′,3′-cGAMP present. Blue blocks indicate size counts as a percentage to overall mass over selected mass ranges. (D) Mass photometry of STING exposed to 2′,3′-cGAMP. **p-value < 0.01; ****p-value < 0.0001 via one way ANOVA test generated with GraphPad Prism.

### Surface Plasmon Resonance (SPR)

To confirm whether CXL affected hSTING in the subfemtomolar range, SPR studies were initiated to determine binding affinities of CXL and endogenous ligands relative to our His-hSTING CTD construct. We performed a control experiment by measuring 2′,3′-cGAMP’s binding affinity for hSTING CTD using an S-Series CMD5 chip. A *K*_D_ for 2′,3′-cGAMP hSTING CTD was obtained () and determined to be 3.45 nM with a *k*_on_ of 1.54 × 10^6^ ± 3.1 × 10^4^ (1/Ms) and a *k*_off_ of 5.93 × 10^–3^ ± 6.6 × 10^–5^ (1/Ms), compared to the *K*_D_ of 3.79 nM reported via ITC.^7^ The *K*_D_ of cyclic di-GMP (c-diGMP) was determined to be 4.78 ± 1.65 μM (). These results are in agreement with our molecular modeling prediction of 2.4 nM *K*_D_ for 2′,3′-cGAMP and 6.4 μM *K*_D_ for c-diGMP (see ). We determined the binding of CXL to hSTING CTD to be 430 ± 140 nM (, Figure 5A). We also examined CXN, which resulted in weaker affinity binding to hSTING CTD of approximately 637 nM (; Figure 5B). This result for CXL was puzzling given the obvious subnanomolar effect of CXL as shown in the *in cellulo* results. Thus, we attempted to obtain SPR data for the interaction between low concentrations of CXL (picomolar and below) and the hSTING CTD bound to an SPR chip. Unfortunately, the RU differences were small compared to instrumental noise, which accentuates the sensitivety limit of the SPR instrument.

**Figure 5:**
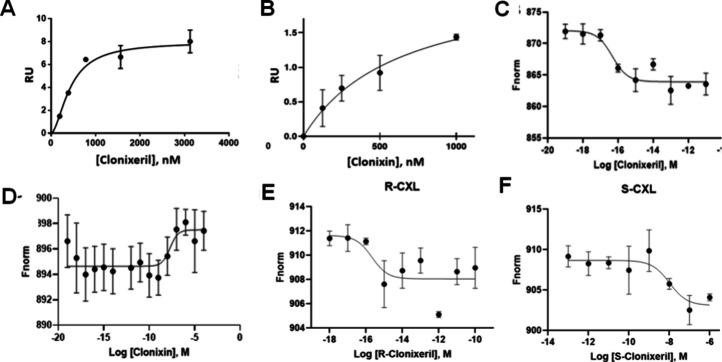
Surface plasmon resonance (SPR) and microscale thermophoresis (MST). (A) SPR analysis for clonixeril interaction from 187 nM to 3 μM without 2′,3′-cGAMP present using STING CTD; (B) SPR analysis for clonixin interaction from 1 nM to 1 μM without 2′,3′-cGAMP present using STING CTD; (C) MST analysis for clonixeril; titration shown is from 100 zM to10 pM to; *N* = 3; *p*-value for shift < 0.0061; (D) MST analysis for clonixin; titration shown is from to 100zM to100 μM; *N* = 3; *p*-value ns; (E) MST analysis for R-clonixeril; titration shown is from 1 aM to 100 pM; *N* = 3; (F) MST analysis for S-clonixeril; titration shown is from 100 fM to 1 μM; *N* = 3.

### Microscale Thermophoresis (MST) Studies

Next, we developed MST protocols that can reliably measure in the subfemtomolar concentration range by leveraging the formation or disruption of presumed oligomer structures. Small molecule affinities at subpicomolar concentrations are typically difficult to determine by MST due to the inability of the detector to measure differential movement of proteins when largely disproportionate ratios of protein to ligand are involved.^33^ We developed a protocol that involves titrating hSTING CTD in the presence of 2′,3′-cGAMP with a potential hSTING antagonist. We first performed a control experiment by titrating hSTING CTD with 2′,3′-cGAMP. This experiment produced a *K*_D_ of 4.00 nM compared to a reported *K*_D_ obtained by ITC of 3.79 nM.^7^ We then titrated CXL into a STING/2′,3′-cGAMP mixture, which presumably functions as a target for analyte interactions with oligomerized STING protein. This strategy provided the sensitivity needed to perform repeatable MST measurements with statistically significant thermal mobility. We determined that CXL possessed a subfemtomolar EC_50_ (Figure 5C, EC_50_ < 1fM). Generally, an IC_50_ correlates well with a *K*_D_ when a small molecule competes for binding with a natural substrate for a target protein.^34^ We use EC_50_ here, though, because we contend that it is unlikely that simple binding is solely responsible for the activity that we observe at very low concentrations (*vide infra*). Notably, CXN, the carboxylic acid precursor of CXL, gave an EC_50_ of approximately 500 nM (Figure 5D). This is consistent with the lower affinity interaction of CXN as measured by SPR (, Figure 5B). Based on our results with CXL and CXN, we synthesized a series of analogues in order to investigate structure activity relationships (SAR) of this chemotype (). Through these experiments, we identified a closely related analogue, mefenamic acid glycerol ester (Figure 1A), which surprisingly gave no indication of activity at any concentration below 1 μM by MST, despite the fact that its chemical structure is nearly identical to CXL (). This result highlights the fact that an SAR was established for CXL analogues in spite of the likelihood that we are not measuring a simple binding event at low concentrations.

### Measurement of the Interaction between CXL and hSTING CTD via Isothermal Calorimetry

In order to further explore the interaction between CXL and hSTING CTD, we employed Isothermal calorimetry (ITC) to measure the heat evolved accompanying CXL’s effect on SUMO-hSTING^WT^ CTD. Given that we used a SUMO-hSTING CTD construct for these studies, we first attempted to reproduce the literature dissociation constant for the binding of c-diGMP to STING’s CTD by ITC. In Figure 6A, we show the ITC data we obtained. Our ITC result for c-diGMP (*K*_D_ = 2.17 μM) is consistent with the value (3.70 μM) previously reported.^16^ In Figure 6B, we show that the interaction of CXL with the hSTING CTD produces essentially the same amount of heat as c-diGMP at approximately the same final concentration, 400 μM (CXL) vs 500 μM (c-diGMP). In Figure 6B, we also find that the binding isotherm is far from steady state or equilibrium, and thus, we could not obtain reliable thermodynamic parameters. Nonetheless, a significant amount of heat was released in the process of CXL interacting with the hSTING CTD. On the other hand, we were most interested in ITC measurements taken at femtomolar concentrations, given the biophysical experiments and *in cellulo* studies already described. Initial experiments treating the hSTING CTD with femtomolar concentrations of CXL were unsuccessful, likely due to the insensitivity of the instrument. Thus, we resorted, as we had before in our MST studies, to competition experiments in which 2′,3′-cGAMP was introduced into the reaction vessel along with the SUMO-hSTING CTD. In Figure 6C we show titration of 400 μM CXL into 40 μM SUMO-hSTING CTD in the presence of 20 μM 2′,3′-cGAMP. This experiment produced similar amounts of heat as CXL alone (Figure 6B). A blank injection of aqueous DMSO to match the highest concentration of DMSO in CXL was titrated into 40 μM SUMO-hSTING CTD in the presence of 20 μM 2′,3′-cGAMP (Figure 6D) to serve as a negative control, indicating that heat present in Figure 6C is the result of the presence of CXL and not due to the DMSO or the interaction of DMSO with 2′,3′-cGAMP. Comparable results to those depicted in Figure 6D were obtained when 2′,3′-cGAMP was absent from the experiment (data not shown). In Figure 6E, 1.5 fM CXL was titrated into 40 μM SUMO-hSTING CTD in the presence of 20 μM 2′,3′-cGAMP. This resulted in a significant evolution of heat in spite of the exceedingly low concentration of CXL.

**Figure 6:**
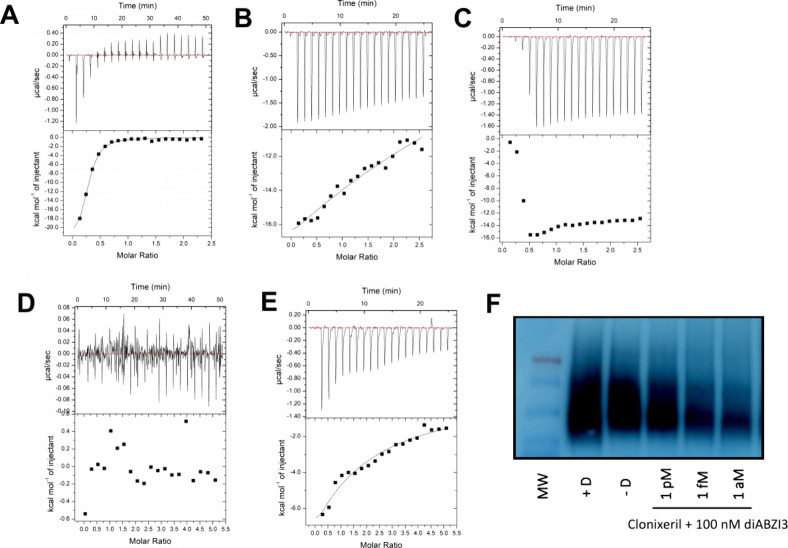
Isothermal Calorimetry of SUMO-hSTING^WT^ CTD and Clear Native PAGE; ITC analysis performed in HBS-P buffer. (A) Binding of 500 μM c-diGMP to 40 μM hSTING CTD. *K*_D_ = 2.17 μM; Δ*H* ^0^ = −2.46 × 10^4^ cal/mol; Δ*S* ^0^ = −56.5 cal/mol/deg. (B) Binding of 400 μM clonixeril to 40 μM hSTING CTD. (C) Binding of 400 μM clonixeril to 40 μM hSTING CTD in the presence of 20 μM 2′,3′-cGAMP. (D) Blank injection of DMSO into 40 μM hSTING CTD in the presence of 20 μM 2′,3′-cGAMP. (E) Binding of 1.5 fM clonixeril to 40 μM hSTING CTD in the presence of 20 μM 2′,3′-cGAMP. (F) Clear native PAGE of HEK293S cells treated with various concentrations of clonixeril, followed by treatment with 100 nM diABZI3.

### CXL Inhibits hSTING Oligomerization As Demonstrated by Clear Native PAGE

To determine whether the results we observed in the DLS experiments could be recapitulated *in cellulo*, where full-length hSTING is present, we used HEK293T cells transfected with WT hSTING (referred to herein as HEK293S cells) to perform hSTING clear native PAGE experiments. Clear native PAGE is required because Coomassie Blue has been shown to disrupt STING oligomerization.^6^ Thus, HEK293S cells were harvested following treatment with 100 nM diABZI3 for 2 h, following a 1 h treatment of varying concentrations of CXL. Our results demonstrate that CXL reduces hSTING oligomerization at concentrations down to attomolar levels with an inverse dose response (Figure 6F). An inverse dose response is consistent with prior observations.

### Clonixeril Enantiomers

It had not escaped our attention that the glycerol “tail” in the ester linkage of CXL, imparts chirality to CXL. Accordingly, we synthesized the two enantiomeric forms of CXL (). Interestingly, in our MST assay, the R-enantiomer shows subfemtomolar activity (Figure 5E), whereas the S-enantiomer seems to show an effect at higher concentrations (Figure 5F). We are currently investigating how the individual enantiomers behave *in cellulo.* In a preliminary study, the R-enantiomer, the S-enantiomer, and the racemic mixture were tested against HEK293S cells for which the hSTING pathway had been activated using diABZI3 and p-IRF3 was the measured end point by Westen blot (). Interestingly, in this experiment, the R-enantiomer is agonistic at 100 fM, and the S-enantiomer exhibits no effect at 100 fM, but it is the racemic mixture that exhibits potent antagonistic behavior at 100 fM. Interestingly, CXN appears to enhance the activity of diABZI3 (100 nM) at a concentration of 100 fM.

## Discussion

We have demonstrated that CXL has unprecedented potency as an hSTING antagonist while also exhibiting weak agonistic activity. In other words, it is a partial agonist. As it turns out, Ergun et al. have shown that unlike 2′,3′-cGAMP, c-di-GMP activates the STING pathway by promoting protein oligomerization without complete dimer angle closing.^35^ They also demonstrated that, at submicromolar concentrations, c-di-GMP inhibits the action of 2′,3′-cGAMP, with an approximate IC_50_ of 800 nM, meaning that c-di-GMP is also a partial agonist. Based on cooperativity observed for STING activation by c-di-GMP (Hill coefficient = 2.6), Ergun et al. proposed that c-di-GMP bound STING could oligomerize with apo-STING, and this could result from the fact that they are conformationally similar (partially closed vs open). In fact, as it turns out, both conformations contain relatively closed dimer angles (*vide infra*). Ergun et al. proposed that hetero-oligomerization increases the rigidity of the STING cyclic-dinucleotide binding site, which subsequently increases the affinity of STING for available c-di-GMP and gives rise to the observed cooperativity. Against that backdrop, we suggest that CXL, as a partial agonist, functions in a fashion similar to that of c-di-GMP but is a more potent antagonist by many orders of magnitude.

Recent cryo-EM studies reported by Liu et al. demonstrate that even full-length apo-STING itself is oligomerized (Figure 1C) to an extent and a manner that is very different from holo-STING (Figure 1C) and that full length apo-STING possesses closed dimer angles relative to X-ray structures of apo-STING CTD.^16^ Based upon the hetero-oligomerization described above involving the association of c-di-GMP bound STING with apo-STING, we speculate that CXL bound STING also interacts with apo-STING in its oligomerized state, and this serves to maintain autoinhibition. We suggest that this effect is nonstoichiometric, and only an exceedingly small number of CXL molecules are needed to stabilize the numerous oligomerized apo-STING molecules, and hence, extremely low doses are needed to affect this type of antagonism. To further support this hypothesis, we have shown by DLS that CXL can prevent the hSTING CTD from forming high order oligomers *in vitro* upon simultaneous treatment with 2′3′-cGAMP, which is consistent with the model that CXL can prevent downstream oligomerization *in cellulo* caused by 2′,3′-cGAMP. Finally, it is unlikely that CXL can reverse STING oligomerization downstream because this state is ultimately stabilized by disulfide cross-linking. This would explain why in our Western blot experiments involving p-hSTING formation using 2′,3′-cGAMP as the activator (Figure 3B), CXL has no effect if HEK293 cells are treated with 2′,3′-cGAMP prior to treatment with CXL. In that case, disulfide stabilized oligomers would not easily be disrupted. Finally, although we do not fully understand why CXL seems to exhibit two distinct binding affinities, we suspect that CXL and c-di-GMP share a common mechanism by which this occurs. For reasons yet to be determined, at concentrations in the micromolar range, they both behave as STING activators by presumably inducing productive oligomerization.

Given the extreme potency of CXL, as demonstrated by DLS, Mass Photometry, MST, and ITC techniques (all of which were applied to hSTING CTD; Figures 4,5,6A-E) and Clear Native PAGE, applied to full-length hSTING using HEK293S cell lysates (Figure 6F), it is unlikely that simple competitive binding of CXL to STING is sufficient to explain its extraordinary effects. It is noteworthy that our very first experiments using differential scanning fluorimetry resulted in a negative thermal shift. That fact suggests two things: (a) there is a protein ligand interaction taking place or there would be no thermal shift; (b) it is not a simple binding event, otherwise the shift would have been positive because the protein would have been stabilized against its thermal denaturation. Finally, it is very unlikely that all of our observations are the result of an artifact since if this was the case, the artifact would have to be repeated across multiple types of *in vitro* and *in cellulo* experiments. To conclude, it is worth noting that our focus on the hSTING CTD for biophysical studies is substantiated by a study from Yin et al. illustrating the importance of the CTD in the hSTING oligomerization process initiated by c-diGMP.^36^ We have, in fact, observed hSTING CTD oligomerization in the presence of c-diGMP using DLS. What is clear from our clear native PAGE experiments with HEK293S cells and DLS experiments using hSTING CTD is that CXL affects hSTING oligomerization, a necessary event for downstream signal transduction. This effect on oligomerization may be dependent on an initial binding event.

We find it intriguing that our ITC experiments have shown that when SUMO-hSTING CTD (pretreated with 2′,3′-cGAMP) is exposed to CXL, a significant amount of heat is released even at a CXL concentration of 1.5 fM (Figure 6E). This result demonstrates, at the very least, that a strong interaction has taken place between CXL and the hSTING CTD consistent with our MST observations. This experiment alone does not provide an indication of exactly what that interaction is. That will hopefully be revealed by Cryo-EM studies. Nonetheless, it is astonishing that measurable heat is released at such a low analyte concentration. Moreover, it is unlikely that the heat release is due to a simple binding event, because we did not obtain an isotherm that is consistent with equilibrium binding thermodynamics. Again, it is tempting to speculate that CXL may engage STING in an initial simple binding event followed by a subsequent change in oligomerization state of STING. It is noteworthy that fairly large amounts of heat can be evolved during an aggregation event.^37^

In Scheme 1, we provide a graphic representation of a possible mechanism that ties together all of the data and conjectures described above. 1. Observation: When we treat HEK293 cells with 2′,3′-cGAMP first, i.e., prior to treatment with CXL, little or no antagonism is observed. Explanation: This observation is consistent with the accepted mechanism for STING activation by 2′,3′-cGAMP in which the disulfide bond formation in the oxidizing environment of the ER and Golgi causes the transduction to be irreversible (Scheme 1A). 2. Observation: When we treat THP-1 cells with high concentrations of CXL alone, we observe agonism (Figure 2A). Explanation: CXL is a partial agonist like c-di-GMP, which causes partial closure of the STING dimer angles. We suggest that CXL, the glycerol ester of clonixin (CXN), may do the same at high concentrations (Scheme 1B). Moreover, we propose that the closely related compound CXN (the carboxylic acid) only engages in the process shown in Scheme 1B. CXN may be a weak agonist like CXL enhancing the activity of diABZI3 in our pIRF3 assay (). Moreover, it exhibits an inflection point by MST in the nanomolar range compared to CXL (Figures 5C and D) 3. Observation: When we treat HEK293 or THP-1 cells with low concentrations of CXL prior to 2′,3′-cGAMP, we see potent antagonism. Explanation: Here we propose that CXL is binding to apo-STING, which is in its oligomerized autoinhibited state, but at low concentrations, an unproductive oligomerization can occur when a single CXL occupied holo-STING molecule interacts with apo-STING nonstoichiometrically (Scheme 1C). This becomes a relatively stable inhibited state that cannot be reversed with 2′,3′-cGAMP. It is likely that partially closed holo-STING is stabilized by partially closed apo-STING, and Ergun et al. have provided cryo-EM some evidence for this hypothesis.^6,35^ On the other hand, it is also likely that there is a profound structural change in the partially occupied apo-oligomers. Our ITC experiments, which granted were done using STING-CTD, support this because as is shown in Figure 6E, every new injection of clonixeril releases from −7 to −2 kcal/mol of heat over the first 15 injections (out of 19) when 1.5 fM clonixeril is titrated into 40 μM hSTING CTD in the presence of 20 μM 2′,3′-cGAMP. We interpret this to mean in the ITC experiment that STING-CTD oligomers preformed with 2′,3′-cGAMP are changing oligomerization state in an exothermic manner with clonixeril acting nonstoichiometrically. 4. Why the inverse SAR? Let us assume in Scheme 1C, which depicts the inhibited state, the concentration of CXL is 1 fM. The 2′,3′-cGAMP signal is knocked down because 2′,3′-cGAMP cannot disrupt the oligomers partially occupied by CXL. Note: in Scheme 1C the oligomers exhibit both *cis* and *trans* configurations with partial occupancy of CXL. Now let us assume that we have CXL at 1 pM concentration. Now the process shown in Scheme 1B begins to compete with the process depicted in Scheme 1C. 2′,3′-cGAMP may also compete with the process shown in Scheme 1B, either way we would observe reduced antagonism relative to CXL at 1 fM, which suggests an inverse dose response as seen in Figures 2B, 2C, 3B, 6F. Notably, this paradoxical behavior has previously been observed for receptor–ligand interactions, but not for STING antagonists.^38,39^ The reported inverse or inverted dose response can take the form of a part of an inverted U-shaped curve (also known as a bell-shaped curve). In fact, when we have observed just such a curve when we test a broader concentration range () instead of just the lower concentrations as shown in Figure 2B.

**Scheme 1:**
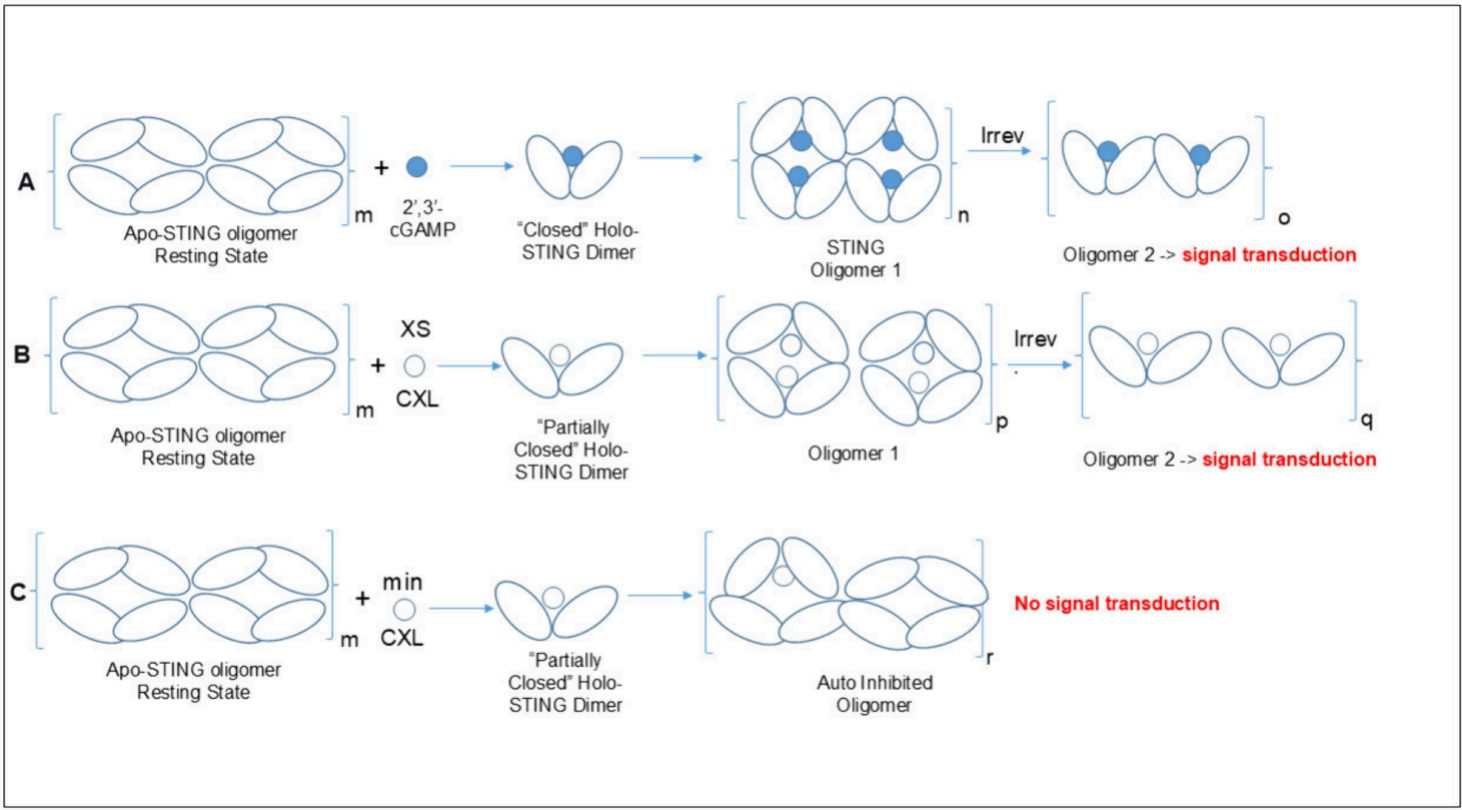
(A) Proposed Mechanism for Activation of STING by Its Endogenous Ligand, 2′,3′-cGAMP.^6^ (B) Proposed Mechanism for the Agonist Activity of CXL Observed at Micromolar Concentrations. (C) Hypothetical Mechanism for Subpicomolar Antagonist Activity of CXL^a^

a Note that a conformational change in the C-terminal tail (not shown) plays a role in exposing the protein oligomer surface upon binding of the ligand; not shown here for clarity. Clonixeril (CXL); Excess (XS); min implies picomolar or lower concentrations.

We reiterate that we have observed the effects of CXL below 1 fM. A simple calculation shows that at 1 fM, the number of molecules per THP-1 cell in our luciferase reporter assay is approximately 1–3 molecules per cell (see ). Whereas, the number of STING molecules per cell is estimated to be between 300 and 1000.^40^ This ratio is perhaps a cause for concern, but given the significant interactions of the STING pathway with the cellular immune response, it is reasonable to expect that transformations in this pathway (both positive and negative) will result in intercellular signal transduction. This could occur through secondary messengers (e.g., presence or absence of pIRF3), paracrine signaling of cytokines, or perhaps juxtacrine signaling from cell-to-cell contact. Juxtacrine signaling through gap junctions or other cell-to-cell contact mechanisms could be very intriguing as it is known 2,3-cGAMP travels from cell to cell in this manner.^24^ It is conceivable that intercellular transport of CXL could increase the probability of its interaction with STING. In other words, perhaps CXL itself does not need to interact with each cell and STING molecule to elicit this overall response, it would only need to interact with a “quorum” of cells that would in turn amplify that information for the local cellular population. Interestingly, against this framework, STING itself is known to engage in intercellular trafficking through a mechanism involving autophagy.^41^

Lastly, we note that CXL may be used as an oral drug in a clinical setting. We have shown that strikingly, it undergoes relatively slow hydrolysis at a pH of 1.0 () and would be stable within the acidic environment in the human stomach. It has already been shown that the oral administration of CXL in rats reduces inflammation. A dose of 30–300 mg/kg CXL in rats has anti-inflammatory effects without producing significant ulcerogenic effects.^42^ We speculate that the findings we describe here could provide a novel breakthrough for the treatment of autoimmune diseases driven by the modulation of STING activity. Should CXL itself not prove to be useful in a clinical setting, it could serve as a lead compound for further optimization. We have already synthesized over 40 CXL analogues that demonstrate an SAR. They are currently being tested in our lab and the laboratories of our collaborators. See for EC_50_’s of CXL and 16 of these analogues measured by MST with their chemical structures shown.

## Conclusion

We have demonstrated that CXL is an extremely potent antagonist of the hSTING^WT^ receptor and exhibits potencies not previously described for small molecule enzyme inhibitors or receptor antagonists, i.e., attomolar (10^–18^ M) levels. Two different hSTING activators (2′,3′-cGAMP and diABZI3) have been employed to compete with CXL, and similar results were obtained. Cell-based experiments were conducted in different laboratories in separate locations and by different personnel, and the results were the same.

In this article, we propose a mechanism of action for CXL’s modulatory effect on the STING pathway. We contend that the proposed mechanism is consistent with our data, where we have shown that CXL modulates STING oligomerization both *in vitro* and *in cellulo*. However, the precise mechanism at the atomistic level of detail for CXL’s unprecedented potency as a STING antagonist will have to await Cryo-EM studies.

## Experimental Section

### Materials

THP1-Dual KI-hSTING-R232 cell lines were purchased from Invivogen (USA). HEK293 cells were purchased from the American Type Culture Collection (ATCC). HEK293T cells were purchased from American Type Culture Collection (ATCC) and transfected with WT hSTING commercially available plasmid (pUNO1-; Invivogen); termed HEK293S cells. Roswell Park Memorial Institute (RPMI) 1640 media containing 25 mM 4-(2-hydroxyethyl)-1-piperazineethanesulfonic acid (HEPES) buffer, Dimethyl sulfoxide (DMSO), SyproOrange and 2 mM L-glutamine were purchased from Thermo Fisher Scientific (USA). Normocin^TM^, Zeocin^TM^, blasticidin penicillin-streptomycin antibiotics were purchased from Thermo Fisher Scientific (USA). Fetal bovine serum (FBS) was purchased from Gibco (USA). CXL was synthesized in house, as described in the . Mefenamic Acid Glycerol Ester was synthesized as described in the . Ultrapure Milli-Q water was given to us by the University of South Florida Geology Department (USA). QUANTI-Luc^TM^ luminescence assay reagent, 6X-His tagged hSTING^WT^, R232 variant, CTD protein was purchased from either Invivogen (USA) or Cayman Chemicals (USA). A Glowmax luminometer was obtained from Promega (USA). The Biacore T200 instrument was obtained from GE Healthcare (USA). 2′3′-cGAMP were purchased from Cayman Chemicals (USA). For His-SUMO-TEV-hSTING CTD see the Protein Production and Purification Methods.

## Computational Methods

### Protein Preparation for Computational Studies

Protein model systems of hSTING CTD variants are prepared using the Schrödinger, Inc. software suite. Protein structure coordinates were obtained from the Protein Data Bank (PDB). Models were generated from PDB entries: 4LOH (hSTING-CTD, H232 allele, 2′3′-cGAMP bound), 4LOI (hSTING-CTD, H232 allele, 2′,2′-cGAMP bound), 4EMT (hSTING-CTD, WT allele, c-di-GMP bound), 4EMU (hSTING-CTD, WT allele, apo structure), 4KSY (hSTING-CTD, WT allele, 2′3′-cGAMP bound), and 4F5W (hSTING-CTD, HAQ allele, apo structure).

### Molecular Dynamics for Virtual Screening

MD simulations were performed with the GPU accelerated Desmond MD program (available from from Schrodinger, Inc.) on two Nvidia GeForce GTX 1080 Ti video cards. A cubic simulation box was created extending at least 10Å from the protein with imposed periodic boundary conditions using TIP3P waters as solvent. The OPLS-3 all-atom force field was then applied to all of the atoms. Simulations were run at a temperature of 310 K and a constant pressure of 1 atm. All systems are energy minimized, followed by multiple restrained minimizations to randomize systems before equilibration and final simulation. Production MD is performed on all systems for 250 ns. Final system equilibration is determined by the observation of the asymptotic behavior of the potential energy, Root Mean Square Deviation (RMSD), and Radius of Gyration (Rg) profiles and visual inspection of trajectories guided by Root Mean Square Fluctuation (RMSF) profiles.

## Computational Docking

After equilibrium is determined, a hierarchical average linkage clustering method based on the RMSD was utilized to determine an average representative structure for the equilibrated hSTING systems. The representative structure is then used for consensus docking incorporating four complementary docking methods available in the Schrödinger, Inc. software suite: SP and XP rigid receptor docking, Induced Fit Docking, and Quantum Polarized Ligand Docking.

As a check for the placement of the GLIDE grids used in the docking studies and for further analysis of the binding cavity for the CDN binding site, Schrödinger’s SiteMap program was employed. SiteMap searches the protein structure for likely binding sites and highlights regions within the binding site suitable for occupancy by hydrophobic groups, hydrogen-bond donors, acceptors, or metal-binding functionalities of the ligand. All ligands were prepared using the program LigPrep and the OPLS-3 all-atom force field was applied to all ligand atoms.

## Rigid Receptor Docking

Rigid docking simulations were performed by the docking program GLIDE (Schödinger, Inc.). GLIDE uses a GlideScore fitness function based on ChemScore for estimating binding affinity but includes a steric-clash term, adds buried polar terms to penalize electrostatic mismatches, and modifies other secondary terms.

## Cellular Assays

### THP1 Luciferase Assays

#### STING THP1 Reporter Assay of IRF3 Promoter with Clonixeril as Agonist

QUANTI-Lucluminesence Dual reporter THP1 cell assay was prepared according to the manufacturer’s instructions (Invivogen, San Diego, CA, USA). A 10 mM stock solution of CXL in 100% DMSO was diluted with ultrapure Milli Q water to make 3, 5, 10, 15, 20, 30, 40, 50 μM samples. The vehicle control contained blank cell medium treated with 0.1% DMSO. 20 μL sample/well of CXL or dilute DMSO (vehicle control) was added to a white, 300 μL, sterile 96 well plate. 180 μL of reporter cells were then plated at 500,000 cells/mL and treated for 10 h instead of the 18–24 h incubation suggested by the manufacturer. These cells were resuspended in media with phenol red but without antibiotics or phosphate-buffered saline (PBS), and these two additives were found to cause statistically significant changes to results. All samples contained a final DMSO concentration of 0.1%. This concentration was found to be nonlethal to THP-1 cells in a cell viability assay. The expression of Lucia luciferase was quantified by measuring the luminescence and evaluated in triplicate. Data are average luminescence changes shown as relative luminescence after subtraction of the background luminescence of vehicle-treated cells. A Glowmax luminometer with an injector was used for the measurement of luminescence in the luciferase assay. After the 10h incubation period, 20 μL/well of cell culture supernatant was transferred to a fresh well plate. A single injector added 50 μL of detection reagent per well, and immediately measured luminescence using a 4s incubation time integrated over 1 s.

### STING THP1 Competition Assay of IRF3 Promoter with 2′,3′-cGAMP as the Activator

The standard procedure from the vendor (Invivogen) for QUANTI-Lucluminesence Dual reporter THP1 cell assay was modified for the competition of CXL with 2′,3′-cGAMP. Thus, an 18–20 h incubation period, media with phenol red, cell density of five hundred thousand cells/mL, and 4 μM 2′,3′-cGAMP as the STING pathway activator were employed. A study was performed to determine that five hundred thousand cells/mL was the optimal cell density. An optimization study was performed to determine the maximum DMSO percentage so as not to impact the viability of the cells. The DMSO was kept at 0.1% or lower, as this was the best concentration to keep the compound in solution and also not affect the viability of the cells. The two controls used in this experiment were media control, which was the standard media with no additives, and the vehicle control was the same percentage DMSO as in the experimental wells with media to establish a baseline. 2′,3′-cGAMP was tested at a variety of concentrations. The chosen concentration was 4 μM. The cells were tested with lipofectamine and compared to wells prepared without lipofectamine. There was no significant difference between the two measurements; it was hypothesized that this is because THP-1 cell lines are known to be permeable to nucleotides like 2′,3′-cGAMP. The optimal incubation time for the cells tested was from 1 h post the addition of 2′,3′-cGAMP up to 24 h. post the addition of 2′,3′-cGAMP. The optimal time for CXL occurred at 10 h. which included a 1 h preincubation time with the compound before the addition of 2′,3′-cGAMP.

### STING THP1 Inhibition Assay Using diABZI3 as the Activator

The cells were grown in RPMI 1640, 2 mM L-glutamine, 25 mM HEPES, 10% heat-inactivated fetal bovine serum, 100 μg/mL Normocin, and Pen-Strep (100 U/mL–100 μg/ mL). For selection, the cells were passaged with and without the addition of antibiotics (10 μg/mL Blasticidin and 100 μg/mL Zeocin) to the growth medium every other passage. Once the cells were confluent, they were pelleted and suspended in test medium containing: RPMI 1640, 2 mM L-glutamine, 25 mM HEPES, 10% heat-inactivated fetal bovine serum, and Pen-Strep (100 U/mL–100 μg/mL). The cells were counted in a cell counter to obtain a cell density of 1 × 106 cells/mL of the test media. The cells were plated (25 μL) in a 384-well Greiner plate (Cat. No. 781098). Compounds were generally dosed at final concentrations of 40, 20, 10, 5, 2.5, 1, 0.5, 0.25, 0.01, and 0.05 μM (1% DMSO final). After 1 h of incubation at 37 °C, the solution contained 50 nM diABZI3. diABZI3 was added to all of the wells containing compounds and the control wells. The negative control wells contained 1% DMSO. The cells were then incubated at 37 °C for 24 h. QUANTI-Luc (InvivoGen) reagent was then diluted in 30 mL of water, 75 μL was added to each well, and luminescence was read immediately (PerkinElmer Envision 2105). The data were normalized to the DMSO only controls (without diABZI3 or 2′,3′-cGAMP), and percentage activation was calculated based on the diABZI3 or 2′,3′-cGAMP only control. Compounds were dosed in triplicate.

## HEK293 Assays

### HEK293 pSTING Western Blot Analysis Using 2′,3′-cGAMP

HEK293 cells were seeded at 700,000 cells per 60 mm plate confluency and treated 36 h later. The samples were first treated with varying concentrations of CXL for 1 h prior to 2 μM 2′,3′-cGAMP (InvivoGen) for 1 h and 30 min using Escort IV transfection reagent (Sigma Aldrich). Two μM 2′,3′-cGAMP was an optimized value based on a dose–response curve for this experiment. Vehicle control group was treated with Escort IV reagent and DMSO for 1 h prior to 2 μM 2′,3′-cGAMP (InvivoGen) for 1 h and 30 min using Escort IV transfection reagent. DMSO concentration was kept constant across all treatment groups. Total cell lysates were collected and analyzed by Western blotting using antibodies against p-STING, STING, and β-actin. Experiments were repeated three times with comparable results. Data points were analyzed with a one-way ANOVA test using PRISM9 statistical analysis software (GraphPad). A level of *p* < 0.05 was considered statistically significant.

### HEK293 Quantitative Real-Time (qPCR) for IFN-β

HEK293 cells were seeded at 700,000 cells per 60 mm plate confluency and treated 36 h later. The samples were first transfected with varying concentrations of CXL for 1 h prior to transfection with 4 μM 2′,3′-cGAMP (Selleck Chem) for 3 h using Escort IV transfection reagent (Sigma Aldrich). Four μM 2′,3′-cGAMP was an optimized value based on a dose–response curve for this experiment Total RNA was isolated using TRIzol (Invitrogen) according to the manufacturer’s instructions. iScript cDNA Synthesis Kit (Bio-Rad) was used to reverse-transcribe cDNA from 1 μg total RNA in accordance with manufacturer’s protocol. SYBR Green real-time qPCR with IFN-β and GAPDH primers was performed for RQ using group treated with 2′,3′-cGAMP alone as reference.

### HEK293 Immunofluorescence

HEK293 cells were seeded into four-chambered slides (5,000 cells/well.) 36 h later, sample 1 was first treated with vehicle control for 1 h and then transfected with 2 μM 2′,3′-cGAMP (InvivoGen) using Escort IV transfection reagent (Sigma Aldrich) for 1 h and 30 min. Samples 2 and 3 were first transfected with 10 aM and 1 pM CXL, respectively, for 1 h, followed by 2 μM 2′,3′-cGAMP for 1 h and 30 min. Two μM 2′,3′-cGAMP was an optimized value based on a dose–response curve for this experiment. DMSO concentration was kept constant across all treatment groups. Cells were fixed with 4% paraformaldehyde for 15 min and immunostained with p-STING antibody at 4 °C overnight with light agitation. The slides were incubated with Alexa 594 rabbit secondary antibody for 1 h at RT. Subsequently, the slides were stained with Phalloidin conjugated to FITC (488) for 30 min at RT, mounted with solution containing 4′,6′-diamidino-2-phenylindole (DAPI) and imaged on a Fluorescent Microscope.

### His-SUMO-TEV-STING (SUMO-hSTING CTD) Protein Production and Purification

The gene encoding human STING (amino acid 155–343) was synthesized and subcloned into pET28a vector (Gift from Dr. Leemor Joshua-Tor of Cold Spring Harbor Laboratory, Huntington, NY) and expressed as N-terminal 6x His and SUMO duo tagged fusion protein in *E. coli* cell strain BL21­(DE3). Cells were harvested 20 h post induction by 0.5 mM IPTG and resuspended in lysis buffer containing 50 mM Tris, pH 7.5, 300 mM NaCl, 20 mM imidazole, 0.5 mM tris­(2-carboxyethyl)­phosphine (TCEP), 5% glycerol, 0.1% Triton X-100, 1 tablet of EDTA free protease inhibitor cocktail/50 mL lysis buffer. The supernatant of the cell lysate was applied to an XK 16 column (Cytiva) packed with Ni-NTA superflow resin (Qiagen), and the fusion protein was washed out by a linear gradient elution with a buffer composed of 50 mM Tris, pH 7.5, 300 mM NaCl, 500 mM imidazole, 0.5 mM TCEP, and 5% glycerol. The purified protein was analyzed by sodium dodecyl sulfate-polyacrylamide gel electrophoresis (SDS-PAGE) (), flash-frozen in liquid nitrogen, and stored at −80 °C for further use.

## Biophysical Assays

### Dynamic Light Scattering

hSTING^WT^ (His-tagged) protein or His-SumoTEV-hSTING protein was filtered using a 100 kDa centrifuge filter at 12,000*g* for 3 min and solubilized at 200nM in HBS-P (0.01 M HEPES pH 7.4, 0.15 NaCl, 0.005% v/v surfactant P20). Concentration was reestablished via nanodrop using Ext. coefficient for His-SUMO-STING at 24870 M^–1^ cm^–1^. Measurements were made using a Malvern Zetasizer Nano ZS set to 40 measurements at 1 s/measurement and 24 runs. Samples were created using 20 μL analyte with 10 μL protein and 10 μL 2′,3′-cGAMP or buffer into a disposable low volume cuvette. The final concentration of all samples were 50 nM STING and 50 nM 2′,3′-cGAMP with a titrated range of analyte.

### Mass Photometry

Microscope coverslips were used in sample preparation for the mass photometry (Refeyn Two^MP^) experiments, by washing three times with Milli-Q water followed by two isopropanol washes. The coverslip was then dried using an air can. Purified SUMO-hSTING CTD protein stock was diluted to 100 nM in a buffer containing 10 mM HEPES pH 7.4, 150 mM NaCl, 5% glycerol, and 0.5 mM TCEP. Twenty μL of each protein sample (100 nM) were incubated for 120 min with 1000 nM of either 2,3-cGAMP or Clonixeril or both. The control sample contained the hSTING CTD protein only. Two μL of each protein sample was added to the coverslip and loaded onto the instrument. A movie of 60 s was recorded. Data was analyzed using DiscoverMP software (Refeyn Two^MP^).

### Surface Plasmon Resonance (SPR)

SPR was employed for binding measurements using the His-tagged hSTING^WT^ CDN domain. A GE Healthcare Biacore T200 was equipped with a Ni-NTA chip. 16,951 RU of 6X-His tagged human STING was cross-linked via NHS chemistry following injections of 350 mM EDTA and 500 mM NiSO_4_. STING natural substrates and the lead compound were titrated and flowed at 60 μL/min in 1× PBS for 60 s association time followed by a 135 s dissociation. The sensorgrams were analyzed using Biacore T200 Software 3.0 and steady state was measured at 4 s before injection stop, exported into Graphpad, and fit versus concentration using a one site specific binding model to calculate the apparent equilibrium dissociation constant (*K*_D_). Where appropriate, kinetics were measured using a 1:1 Langmuir binding model with *R*_max_ set to local to obtain the association rate (*K*_on_), dissociation (*K*_off_)

### Microscale Thermophoresis (MST)

STING R232 variant (human recombinant, wild-type) protein was solubilized at 200nM in HBS-P (0.01 M HEPES pH 7.4, 0.15 NaCl, 0.005% v/v surfactant P20) along with 100 nM 2′,3′-cGAMP. 100 nM portion of NTA-Atto 488 dye (blue; nitrilotriacetic acid complexed to Ni^2+^ ion) is added and incubated for 1 h at RT covered from light. The resulting mixture was centrifuged at 12,000 x RPM 10 min prior to use. A 1:10 series dilution from 200 μM to 200zM was created using HBS-P with 1% DMSO. The dyed STING/2′,3′-cGAMP mixture was added to each sample in a 1:1 ratio, resulting in a static 100 nM STING, 50 nM 2′,3′-cGAMP, and a concentration range from 100 mM to 100 zM. Samples were incubated for 15 min prior to loading into Monolith NT.115 capillaries and run on NanoTemper Pico instrument. The samples were run again at 30- and 45 min. Detection of the protein was performed using the blue detection channel with excitation power set to 100% and MST set to high allowing 3 s prior to MST on to check for initial fluorescence differences, 35 s for thermophoresis, and 3 s for regeneration after MST off. Analysis was performed using M.O. Affinity Analysis Software with difference between initial fluorescence measured in the first 5 s as compared with thermophoresis at 30 s at 16 different analyte concentrations ranging from 100 μM to 100 zM and exported into Graphpad Prism v.8 using a Log inhibitor versus response for parameter fit. MST Confidence Intervals for clonixeril. From Top 871.7 to Bottom 864.0; logEC_50_ −16.28; Hillslope −1.669; Span 7.723; Degrees of Freedom 5; R squared 0.9033; Sum of Squares 11.55; Sy.x 1.520.

### Isothermal Titration Calorimetry (ITC)

MicroCal ITC200 instrument (Malvern Pananalytical) was used to assess STING and CXL interactions. Both recombinantly purified STING domains (residues 155–343) containing a SUMO tag and CXL were buffer-exchanged and made in the same buffer composition, respectively, to avoid buffer mismatch. The same buffer cocktail was used in all ITC experiments, containing 25 mM HEPES (pH, 7.4), 150 mM NaCl, 5% glycerol, 0.5 mM TCEP, and 5% DMSO. STING protein from −80 °C aliquots was spun down at 12,000 RPM for 5–10 min before each run to remove any aggregates, diluted into the ITC buffer above (either as 40 μM STING alone or as 40 μM STING plus 20 μM 2,3-cGAMP), and loaded into the cell. CXL at various concentrations for different experiments was loaded into the syringe (Hamilton). For the control and reference, an ITC buffer devoid of CXL was used. ITC experiments were conducted at 25 °C using an initial 0.4 μL injection and 19 subsequent injections of 2 μL each at 150 s intervals. Heat of dilution (differential power) of the 19 injections resulting from the binding event was fitted into the nonlinear least-squares equation incorporated in the MicroCal ITC200 analysis software. The *K*_D_ and other thermodynamic parameters were derived from curve fitting using MicroCal software.

### Clear Native PAGE

Invitrogen NativePAGE Sample Prep Kit Catalog number: BN2008, Invitrogen NativePAGE Running Buffer Kit Catalog number: BN2007 and Invitrogen NativePAGE 4 to 16%, Bis-Tris, 1.0 mm, Mini Protein Gels, 10 wells, (Cat. # BN1002BOX). Cells were harvested and CXL was added at varying concentrations for 1 h and followed by 2 h of diABZI3 at 100 nM. To the mammalian cells harvested in 1 mL cell culture, add 0.2 mL lysis buffer containing 10 mM HEPES, pH 7.5,150 mM NaCl, 5% glycerol, protease inhibitor cocktail, and 0.025% digitonin. Cells were lysed by sonicating for two rounds of 15 s each while cooling the sample on ice. Centrifuge the lysate at 20,000*g* for 10 min at 4 °C. Aliquot the supernatant into microcentrifuge tubes. To prepare the sample for loading a total volume of 20 μL NativePAGE including Sample Buffer (4×) 5 μLSTING supernatant, 14.5 μL and Ponceau S-dye: 0.5 μL. 1X NativePAGE Anode Buffer: Add 30 mL of 20X NativePAGE Running Buffer to 570 mL of deionized water. 1× NativePAGE Light Blue Cathode Buffer: Add 10 mL 20× NativePAGE Running Buffer and 1 mL 20× NativePAGE Cathode Additive to 189 mL deionized water. The gel was run at 150 V constant for 110 min. Gel was developed using A Western blot using a PVDF transfer membrane was performed by the eBlot L1 Protein Transfer System.

## Supplementary Material





## Data Availability

All data to understand and assess the conclusions of this research are available in the main manuscript or within the . Analogue compounds pertaining to CXL are under patent consideration and therefore are not available for transfer. The synthetic scheme used for CXL is included in the .

## Notes

Supplemental figures, tables, methods, experimental procedures, synthesis of main compounds and enantiomers, ^1^H NMR data (PDF)
Transparent Peer Review report available (PDF)
