# Supplementary material for: Discovery of Clonixeril as a Sub-Femtomolar Modulator of the Human STING Receptor
Source: ACS Cent Sci. 2025 Jun 6;11(6):994–1008. doi: 10.1021/acscentsci.4c01982 (PMC12203428; doi:10.1021/acscentsci.4c01982)
Supplement: Supplementary file 1 [file oc4c01982_si_001.pdf]

Supporting Information for

Discovery of Clonixeril as a Sub-Femtomolar Modulator of the Human STING Receptor

Robert P. Sparks[1,2,3†], William Lawless[1,2,4†], Anna Kharitonova[1,2,4†], Rainer Metcalf[1†], Jamie Nunziata[1], Grace A. Binder[1], Sauradip Chaudhuri[5], Christine S.R. Gambino[1], Michelle Wilde[1], Linette S. Harding[1], Jaret J. Crews[1], Mansi Gopu[1], Emilia Dalamangas[1], Sarah Lawless[1], Mark Eschenfelder[1], Robert M. Green[5], Elizabeth X. Nompleggi[3,7], Timothy Tran[6], Yan Yang[6], Donna V. Trask[1], Paul R. Thompson[5], Rekha Patel[2,4], Niketa A. Patel[2,4], Wesley H. Brooks[1], Guy Bradley[8], Mildred E. Acevedo-Duncan[1], Alan C. Mullen[3], James W. Leahy[1,4,9], Kenyon G. Daniel[1,10], Wayne C. Guida*[1,6,9]

Affiliations:

[1] Department of Chemistry; University of South Florida, Tampa, FL, USA.
[2] Research Service; James A. Haley Veterans Hospital, Tampa, FL, USA.
[3] Division of Gastroenterology, Department of Medicine; University of Massachusetts Chan Medical School, Worcester, Massachusetts, USA.
[4] Department of Molecular Medicine, Morsani College of Medicine; University of South Florida, Tampa, FL, USA.
[5] Biochemistry and Molecular Biotechnology Department; University of Massachusetts Chan Medical School, Worcester, Massachusetts, USA.
[6] H. Lee Moffitt Cancer Center, Research Institute at the University of South Florida; Tampa, FL, USA.
[7] College of the Holy Cross, *Worcester, MA, USA.*
[8] Tampa Bay Research Institute, St. Petersburg, Florida, USA
[9] Florida Center for Drug Discovery and Innovation, University of South Florida, Tampa, FL, USA
[10] Department of Molecular Biosciences, University of South Florida, Tampa, FL, USA.

* Corresponding Authors: Dr. Wayne C. Guida, Department of Chemistry, University of South Florida, CHE 205, 4202 E. Fowler Avenue, Tampa, FL 33620, USA, wguida@usf.edu

Table of Contents

Supplementary Text 3
MD Simulations in Support of Computational Model Construction 3
Site-Restriction Virtual Screening for Identification of Possible Dimer Complexes 3
Differential Scanning Fluorimetry – Thermal Shift Experiment 4
Clonixeril Demonstrates Limited Hydrolysis under Digestive Conditions 4
Clonixeril affects pIRF3 production only through the STING pathway 4
Calculation of the number of molecules per cell in the THP1 IRF3 luciferase reporter assay 4
Supplementary Methods 5
Synthesis of Clonixeril and its Enantiomers 5
Synthesis of Mefenamic Acid Glycerol Ester 6
Differential Scanning Fluorimetry (Thermal Shift) 6
Solubilization and HPLC Mass Spectrometry of CXL and CXN 6
Phospho-IRF3 Assay 7
Alternative Protocol for STING THP1 Competition Reporter Assay with 2',3'-cGAMP as the Activator and Digitonin as a Permeabilization Agent 7
Supplementary Tables and Figures 9
Table S1. Summary of K_d values for some biophysical data 9
Table S2. Computer Model Comparisons of Literature and Experimental Values 9
Figure S1. Differential Scanning Fluorimetry (Thermal Shift) for Binders of STING. 10
Figure S2. Structures of hSTING (CTD) with Clonixeril bound 10
11
Figure S3. Immunocytochemistry of HEK293 cells showing pSTING. 11
Figure S4. SPR, Clonixeril Stability, Cell Viability, and THP1 STING Knockout Data. 11
Figure S5. SAR for Clonixeril and Analogs 12
Figure S6. MST for Clonixeril and Mefenamic Acid Glycerol Ester 13
Figure S7. Western Blot for HEK293S cells treated with Clonixeril and its enantiomers 13
Figure S8. Purity of His-SUMO-TEV-STING 13
Figure S9. Luciferase Assay Utilizing Monocytic Leukemia (THP-1) cells 14

Supplementary Text

MD Simulations in Support of Computational Model Construction

We employed molecular dynamics (MD) simulations to better understand how STING interacts with endogenous ligands and other potential binding partners. The distance between the α-carbons of both H185 residues at the end of the α2 helices in dimeric $hSTING^{WT}$ CTD was used as a metric for STING activation. Crystal structures of known agonists showed alpha carbon distances in the range of 34 to 38 Å (PDB: 4EMU) for holo structures, whereas apo crystal structures had alpha carbon distances in the range of 47 to 56 Å (PDB: 4KSY, **Table S2**). Initial binding of ligands to $hSTING^{WT}$ CTD near residues Q266 and T267 stabilizes the disordered "lid" region towards β sheet formation, bringing the α2 helices closer together. This prompted generation of two separate docking models (**Figure 1C**) to screen for (a) agonists, compounds that have greater affinity for the holo (2',3'-cGAMP bound structure), and thus stabilize the ordered lid, and (b) antagonists, compounds biasing a more disordered lid conformation.

Our statistical models were compiled based on deviation from known values and internal variance to adjust for docking and simulation error (**Table S2**). ITC, SPR, and consensus docking energetics for the hSTING wild type ($hSTING^{WT}$) native ligands 2'3'-cGAMP and c-di-GMP (**Table S2**) are in close agreement, supporting the validity of our MD equilibrated $hSTING^{WT}$ binding models. Due to the dominance of the WT allele in human populations, the MD equilibrated $hSTING^{WT}$ antagonist (PDB: 4F5Y) and $hSTING^{WT}$ CTD agonist (PDB 4KSY) conformations were taken as the two specific docking models for subsequent STING molecular modeling.

Site-Restriction Virtual Screening for Identification of Possible Dimer Complexes

Current docking programs are restricted to docking and evaluating a single molecule at a time. Due to this, docking algorithms are unable to effectively predict ligands which bind as dimers, such as the binding of DMXAA to mouse STING. To overcome this limitation, we developed a simple docking method that can be used in conjunction with standard virtual screening protocols to assist in identifying potential small molecule dimer-protein complexes. Initially while utilizing the entire protein dimer structure, the ligand is restricted to a monomeric half of the binding site (**Figure S2A**). This procedure, as opposed to an alternate docking protocol involving the protein monomer alone, allows for site electrostatics that are consistent between the whole site and the restricted portion. Then, following the typical virtual screening protocol, the whole site (**Figure S2B**) is employed for ligand docking. To avoid oversimplification of the ligand binding geometry and to test assumptions of ligand and half-site interaction, the next step is to implement an RMSD comparison of the ligand poses for the whole and half site docking runs. If the RMSD between the whole and half site poses is less than the commonly accepted 2 Å cutoff, then the docked ligand evidently prefers a specific region in the binding site and should allow for another stoichiometric equivalent of the molecule to bind into the surplus volume. After review of the initial ligand poses, updated docking grids are generated with the original docked compound and the unoccupied region is subsequently screened with a duplicate ligand (**Figure S2C**). The dimer composite structure could then potentially be linked through a zero-ordered bond connecting the two most proximal atoms. For GLIDE, this type of bond only has an enforced distance constraint, angle and dihedral terms are zero, and do not interfere with the molecular force field. Docking the linked dimer (LD) back into the respective protein conformer will allow the

docking algorithm to properly calculate estimated free energies of binding for the LD-protein complex.

Differential Scanning Fluorimetry – Thermal Shift Experiment

The change in melting point of hSTING^{WT} CTD (**Figure S1)** was observed in 3 scenarios: 1) bound to the endogenous ligand, 2',3'-cGAMP, 2) unbound and 3) bound to ligands chosen by the virtual screening process for their potential to stabilize the active form of the STING molecule by binding to it. A TSA measures a protein's melting temperature (Tm), at which the protein is 50% denatured. The assay quantifies protein denaturation by measuring the increase in fluorescence of a dye that binds to hydrophobic residues exposed by unfolding of the protein.

Clonixeril Demonstrates Limited Hydrolysis under Digestive Conditions

To determine clonixeril's hydrolytic stability and evaluate its potential as an oral drug, an aqueous solution was concentrated for analysis at 20 μM. The sample was analyzed by HPLC using an isocratic gradient of water and methanol and resulted in spectra demonstrating two distinct peaks. The UV spectra of these peaks were compared by diode array and found to be identical with maximum absorbance at 280nm and 343nm. LC/MS demonstrated that the molecular weight of the minor first peak matched the molecular weight of clonixin (M+H)+ and the second major peak matched the molecular weight of clonixeril (M+H)+. Data was generated by measuring the ratio between the two peaks. Hydrolysis of clonixeril to clonixin was measured by HPLC over seven days using a 20 μM aqueous solution. Initial hydrolysis was found to show 3.2% clonixin and 96.8% clonixeril (**Figure S4B**). Most of the hydrolysis occurred within the first two days at an average rate of 0.13% per hour, and then slowed down to an average rate of 0.07% per hour by day seven (**Figure S4B**). The rate decrease correlated with an observable decrease in solution pH as a product of hydrolysis. Further samples solubilized in pH 2.00 phosphoric acid and pH 1.99 hydrochloric acid confirmed that the rate decreases to an average of 0.014% per hour over a seven-day period. Samples solubilized under basic conditions such as 5% sodium bicarbonate resulted in an increase in initial hydrolysis to 11.8% clonixin and 88.2% clonixeril.

Clonixeril affects pIRF3 production only through the STING pathway

Because pIRF3 can be produced from TBK1 via pathways independent of STING (i.e., TLR7 and MDA5/RIG-I), we tested clonixeril in a STING knockout THP-1 Luciferase assay. The assay method was identical to the one reported in the main text hSTING^{WT}. As is shown in **Figure S4D**, we demonstrate that clonixeril is affecting pIRF3 production only through the STING pathway.

Calculation of the number of molecules per cell in the THP1 IRF3 luciferase reporter assay

The volume of the well = 200 μL = 0.2 mL = 2x10^-1mL but cells are transferred in 180 μL of media and the volume of the solution is then brought up to 200 μL.

5x10^5 cells/mL x 1.8x10^-1 mL = 9x10^4 cells per assay well

2.5x10^5 cells/mL x 1.8x10^-1 mL= 4.5x10^4 cells per assay well

The concentration of compound at 1 fM = 10^-15 mmol/mL

1x10^-15 mmol/mL * 2x10^-1 mL per well = 2x10-16 mmol

2x10^-16 mmol x 6.02 x 10^20 molecules /mmol = 12.04 x 10^4 molecules per assay well

At a cell density of 2.5x10^5 cells/mL which = 4.5x10^4 cells per assay well, we calculate 2.68 molecules per cell at 1fM.

At a cell density of 5x10^5 cells/mL which = 1.34x10^4 cells per assay well, we calculate 1.34 molecules per cell at 1fM.

Supplementary Methods

Synthesis of Clonixeril and its Enantiomers

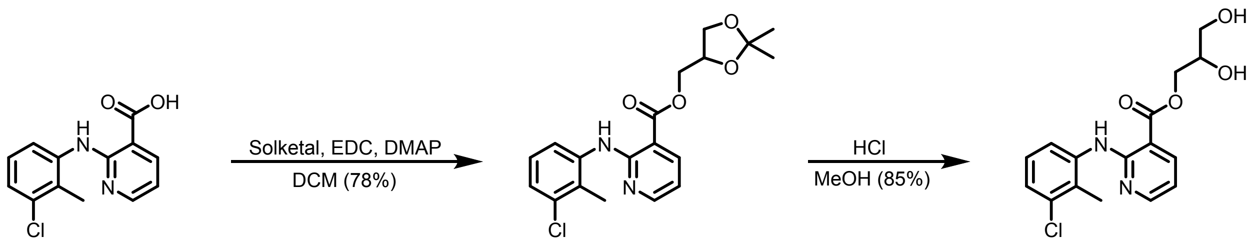

A solution of clonixin (544.3 mg, 2.072 mmol) in dichloromethane (10 mL) was cooled to 0 °C in a round bottomed flask with stirring. Solketal (1.29 mL, 10.4 mmol), EDC (795.1 mg, 4.148 mmol) and DMAP (13 mg, catalytic) were added, and the reaction was stirred at 0 °C for 1 h before warming to room temperature overnight. The reaction was quenched with water (10 mL) then extracted with DCM and the combined organic layers washed with saturated sodium bicarbonate and concentrated on a rotary evaporator to give a gummy residue (609 mg, 78%) that was used without further purification.

A solution of the ketal protected clonixeril obtained above (609 mg, 1.62 mmol) was dissolved in methanol (10 mL) and cooled to 0 °C in a round bottomed flask. A few drops of a solution of 3M HCl in methanol was added, and the reaction was allowed to warm to room temperature overnight. The solution was then concentrated without heat on a rotary evaporator to give a yellow oil that was purified by flash column chromatography (DCM/MeOH 19:1) to give clonixeril (463 mg, 85%) as a colorless oil. ^{1}H NMR (600 MHz, $CDCl_3$) δ 9.75 - 9.83 (m, 1 H), 8.25 - 8.30 (m, 1 H), 8.16 - 8.23 (m, 1 H), 7.73 - 7.78 (m, 1 H), 7.05 - 7.14 (m, 2H), 6.62 - 6.69 (m, 1 H), 4.30 - 4.40 (m, 2 H), 3.97 - 4.04 (m, 1 H), 3.69 - 3.76 (m, 1 H), 3.57 - 3.64 (m, 1 H), 2.31 (s, 3 H) ppm. ^{13}C NMR (151 MHz, CD_3OD) δ 169.3, 156.4, 151.4, 141.2, 138.7, 134.4, 129.0, 126.5, 124.8, 122.2, 113.3, 108.5, 13.6 ppm.

Synthesis of (R)-Clonixeril

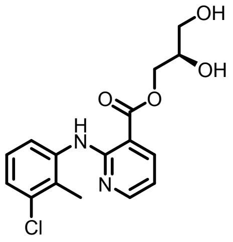

(R)-clonixeril was synthesized using the procedure outlined above for racemic clonixeril, but with commercially available (R)-solketal (obtained from Aaron Chemical) used in place of racemic solketal. (R)-clonixeril had identical analytical data to that obtained above. (S)-clonixeril likewise was prepared using (S)-solketal.

Synthesis of Mefenamic Acid Glycerol Ester

Mefenamic acid glycerol ester was prepared using the procedure described above for clonixeril with the exception of mefenamic acid being employed as a starting material rather than clonixin. Yield 85.0%. ^{1}H NMR (600 MHz, CHLOROFORM-d) δ 9.02 - 9.13 (m, 1 H), 7.82 - 7.91 (m, 1 H), 7.14 - 7.18 (m, 1 H), 7.04 - 7.08 (m, 1 H), 7.00 - 7.04 (m, 1 H), 6.92 - 6.97 (m, 1 H), 6.63 - 6.67 (m, 1 H), 6.55 - 6.59 (m, 1 H), 4.29 - 4.37 (m, 2 H), 3.98 - 4.04 (m, 1 H), 3.69 - 3.74 (m, 1 H), 3.61 - 3.65 (m, 1 H), 2.75 - 3.16 (m, 1 H), 2.35 - 2.70 (m, 1 H), 2.24 (s, 3 H), 2.08 (s, 3 H) ppm. 13C NMR (151 MHz, CHLOROFORM-*d*) δ ppm 168.9, 149.8, 138.5, 138.3, 134.6, 132.6, 131.5, 127.0, 126.0, 123.3, 116.1, 113.8, 110.1, 70.5, 65.3, 63.5, 20.6, 14.0 ppm.

Differential Scanning Fluorimetry (Thermal Shift)

QuantStudio and Protein Thermal Shift Software were used to analyze 2 µM CTD STING protein in 25 µM TBS at pH 8, 2% DMSO and SyproOrange. 57 compounds including CXL were screened at 200 µM to determine melting temperature as compared to controls which were CTD STING protein without 2'3'-cGAMP and CTD STING protein with 200 µM 2'3'-cGAMP as a direct comparison. The T-ramp range was between 25-99 °C within 1 hour.

Solubilization and HPLC Mass Spectrometry of CXL and CXN

A bathtub sonicator was used at room temperature to aid solubilization of CXL. Care was used to avoid heating the solution and causing unwanted hydrolyzation through the addition of vibrational energy supplied from a sonication probe. An isocratic gradient program of mobile phase A (100% water) and mobile phase B (100% methanol) was established on a Shimadzu liquid chromatography system, and the column was initially brought to equilibrium at 10% B. An isocratic gradient program was performed, whereas mobile phase B remained at 10% for the first five minutes and then 10% to 80% gradient from 5 minutes to 35 minutes. A sample was created at 200uM using 100% methanol and 500µL was shot onto Agilent 1260 Preparative HPLC-DAD-MS SQ 6120. Retention times for both peaks were relatively similar. The two peaks were collected, dried using nitrogen, solubilized in acetonitrile, and run on the Agilent LC-MS QTOF 6540.

Phospho-IRF3 Assay

HEK293T cells transfected with $hSTING^{WT}$ cells were seeded in a 6-well plate in DMEM (supplemented with 10% heat-inactivated fetal bovine serum, 1X Corning Penicillin-Streptomycin solution and 0.01mg/mL Blasticidin). Upon reaching ~80% confluence, cells were pretreated with varying concentrations of CXL in FBS-free DMEM for 2 hours. This was followed by treatment with diABZI3 (100nM) for 1 hour. The cells were scraped, harvested by centrifugation at 1000 x g for 3 mins. The resulting pellet is resuspended in 1X PBS with 1X Halt protease & phosphatase inhibitor and 1% NP-40. Cell lysis was performed by probe sonication. The cell lysate is further denatured in 1X SDS loading buffer by boiling for 10 mins and separated by SDS-PAGE (4-20% gel). The separated proteins were electrically transferred to a PVDF membranes separately, which was probed with primary (α-pIRF3, α-IRF3 & α-STING) and secondary (goat anti-rabbit IR dye 680 & goat anti-mouse IR dye 800CW) antibodies. The blots were visualized using a LICOR Image Analyzer. All the experiments were performed at least in duplicate.

Alternative Experimental Protocol for STING THP1 Competition Reporter Assay with 2',3'-cGAMP as the Activator and Digitonin as a Permeabilization Agent

In order to permeabilize relatively impermeable CXL analogs, we developed a digitonin-based assay to be used in conjunction with our STING THP1 competition assay. The protocol is given here.

Reporter Cells
THP-1 Dual KI $hSTING^{WT}$ R232 cells (cat no. thpd-nfis) were obtained from Invivogen (San Diego, CA) and processed as per the manufacturer's instructions. The cells were cultured at 37C in 5% CO2 in complete RPMI media (RPMI 1640 GlutaMAX I supplemented with sodium bicarbonate (2.0 g/L), D-glucose (2 g/L), 10% fetal bovine serum, penicillin (100U/mL), streptomycin (100 µg/mL) and Normocin (100 µg/mL). The cultures were maintained at cell densities between 5 x 10^5 and 2 x 10^6/mL.

Digitonin facilitated STING THP1 Cell assay
Prepared the treatment solutions in 5 mL polystyrene snap cap tubes (labeled A-G). Tube A (negative control) contained 1.5 mL digitonin buffer (DB; 50 mM HEPES pH 7.0, 100 mM KCl, 3 mM $MgCl_2$, 0.1 mM DTT, 85 mM sucrose, and 0.2% BSA) only. Ten mL of DB and 1.25 ug/mL digitonin (stock is 10 mg/mL in H2O) were added to a 15 mL conical polypropylene tube, mixed well, and then 1.5 mL of this solution was aliquoted into tubes B-G. Starting with a Clonixeril concentration of 1 µM in Tube C, 1000-fold serial dilutions were generated (1nM, 1pM, 1fM, and 1 aM). Transferred 5 x 10^5 of freshly harvested THP-1 Dual KI hSTING R232 cells into seven 5 mL snap cap polystyrene tubes labeled 1-7. Pelleted the cells using a Clay Adams Sero-Fuge II centrifuge at 1000 x g for 2 minutes. The media was carefully removed by aspiration. Each pellet was suspended in 1 mL of the treatment solutions (A-G, respectively) vortexed to ensure mixing. The cells were incubated at 37°C for 10 min. Then, as quickly as possible, 10 µL of a 1 mM stock solution of 2'3' cGAMP was added to each tube and vortexed to mix. The cells were incubated at 37°C for 10 min. At the end of the 10 min treatment, 2 mL of complete phenol red free RPMI (10% FCS, 1x penicillin/streptomycin) was immediately added to all tubes and mixed well. The cells were centrifuged for 2 min at 1000 x g. The supernatant from each tube was removed, the cells suspended in 500 µL of fresh phenol-red free complete RPMI media and incubated at 37°C for 4 to 24 h (preferably 6 h).

Detection of IRF3 gene activation
After the 6 or 24 h incubation, the cells in each tube were vortexed and then 20 µL of the cells were transferred into quadruplicate wells of a solid white plate. The detection of luciferase generated by the THP-1 Dual KI hSTING R232 cells was performed using Invivogen's QUANTI-Luc 4 Lucia/Gaussia solutions and the associated "glow" protocol. Using a multichannel pipet, 50 µl of the QUANTI-Luc™ 4 Lucia/Gaussia "Glow" solution (with added stabilizer) was added to each well and gently mixed. The plate was immediately placed in Modulus Microplate reader and the relative light units (RLU) were measured using an integration time of 0.5 sec.

Panels A and B below illustrate the efficacy of the experimental method described (6 h incubation time). The results in Panel A were obtained at Tampa Bay Research Institute by of our collaborator. Panel B results are from experiments done at USF.

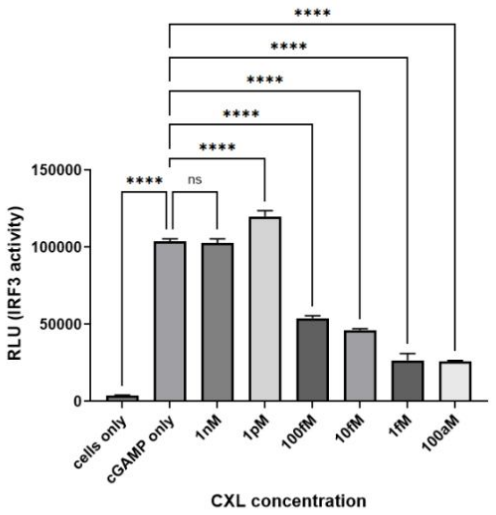

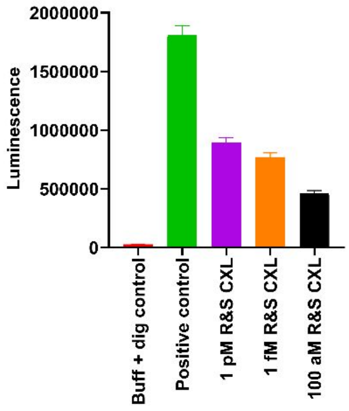

Panel A

Panel B (positive control = 2',3'-cGAMP alone)

Supplementary Tables and Figures

Table S1. Summary of K_d values for some biophysical data

CPD	Technique	K_D
Cyclic-diGMP	SPR –Steady state	4.80 µM
2’,3’-cGAMP	SPR – Steady state	3.56 nM
Clonixeril	SPR – Steady State	430 nM
Clonixin	SPR – Steady State	637 nM
Cyclic-diGMP	ITC	2.17 µM
2’,3’-cGAMP	Thermophoresis	4.00 nM
Clonixin<br>+50 nM 23cGAMP	Thermophoresis	~500 nM
MFE<br>+50 nM 23cGAMP	Thermophoresis	No shift

Table S2. Computer Model Comparisons of Literature and Experimental Values

Structure	Compound	H185 Distance (Angstroms)	H185 Post-MD Distance (Angstroms)	Model K_D (nM)	SPR K_D (nM)	ITC K_D (nM)	Cell EC_{50} (nM)
$hSTING^{WT}$	c-2'3'-GAMP	35.0[a]	37.8	2.4	1.4	3.8[a]	42 (IFNβ mRNA)[a]
	c-2'2'-GAMP	-	43.1	256	-	287[a]	16 (IFNβ mRNA)[a]
	c-di-GMP	53.0[c]	56.5	6377	4776	1210[a]	538 (IFNβ mRNA)[a]
$hSTING^{REF}$	c-2'3'-GAMP	34.7[b]	39.3	784	-	5300[b]	1200 ELISA[b]
	c-2'2'-GAMP	38.4[b]	41.9	236	-	2500[b]	3400 ELISA[b]
	c-di-GMP	52.6[d]	54.1	1300	-	4600[c]	ND (IFNβ Luc)[c]
						4420[d]	ND (IFNβ Luc)[d]

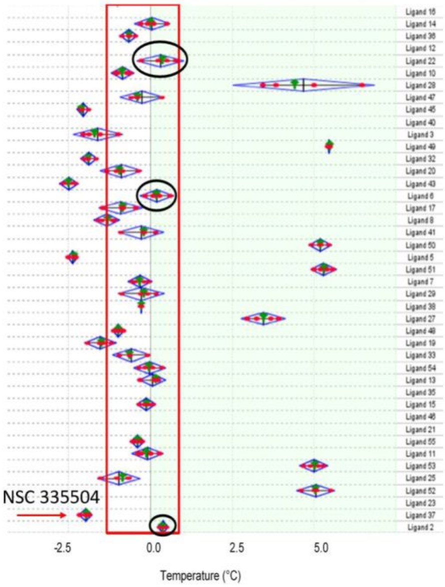

Figure S1. Differential Scanning Fluorimetry (Thermal Shift) for Binders of STING.
Compounds selected (57) from computational virtual screening for analysis at 200 μM in the thermal shift assay are shown here as green dots with blue diamond indicating the temperature range of denaturation. The red box indicates the hSTING CTD control with 200 μM 2',3'-cGAMP at 5.47, 5.19, 5.29, 5.00 5.05 ^{o}C respectively. Black ovals are compounds with significant ΔTm above the hSTING CTD. Clonixeril (NSC 335504) is ligand 37.

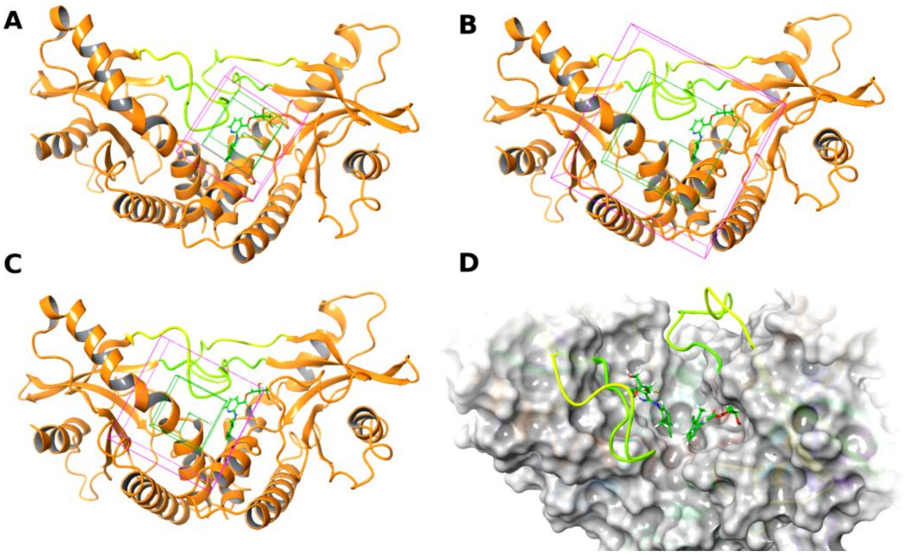

Figure S2. Structures of hSTING (CTD) with Clonixeril bound. Green boxes indicate ligand centroid positional constraint and purple boxes represent all ligand atom positional constraint. Lid region demarcated with yellow-green ribbons. (A) Site-restriction docking method for identifying potential dimeric ligand complexes. Initial docking to monomer unit of binding site is performed with half of the site excluded. (B) Secondary re-docking of ligand is performed with no restrictions. (C) If ligand maintains its pose in both docking simulations, a second copy of the ligand is re-docked to a new grid with the original ligand held in place. The re-docked copy can then link to the initial pose with a zero-order bond, connecting the most proximal atoms, and re-docked once more (D) Antagonist model with two CXL molecules bound shown (molecular surface shown for clarity).

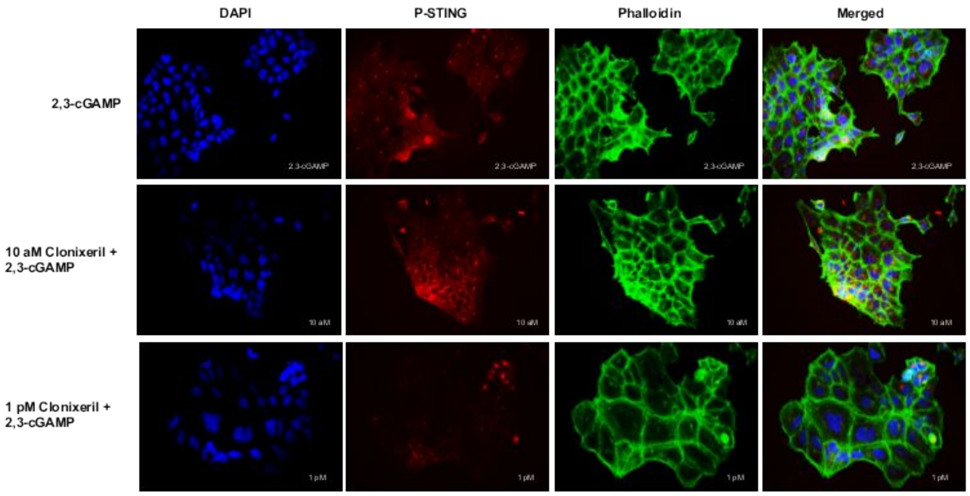

Figure S3. Immunocytochemistry of HEK293 cells showing pSTING. HEK293 cells were treated with 10 aM or 1 pM clonixeril in the presence of 2 µM 2',3'-cGAMP. Cells were incubated with pSTING rabbit primary antibody followed by Alexa 594 anti-rabbit secondary antibody. Cells were stained using phalloidin conjugated to FITC (488) in a solution containing 4',6'-diamidino-2-phenylindole (DAPI). Images were taken using a Fluorescent Microscope.

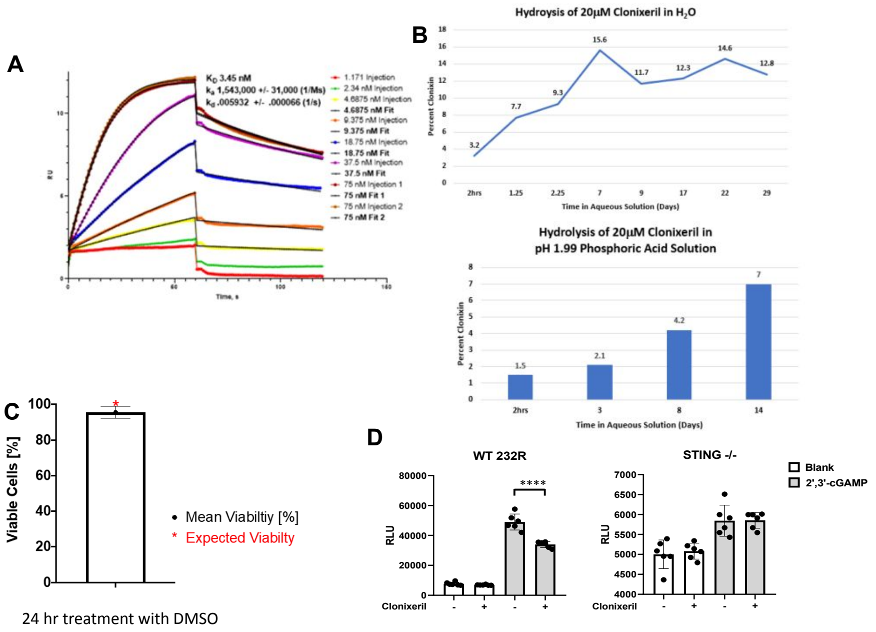

Figure S4. SPR, Clonixeril Stability, Cell Viability, and THP1 STING Knockout Data. (A) SPR analysis for 2',3'-cGAMP interaction using hSTING CTD. (B) Hydrolysis of clonixeril in aqueous solution pH=7 for 28 days and pH=2 for days 14 days. (C)THP-1 cell viability. DMSO concentration 0.1%. (D) Quantification of luciferase assay performed using WT-THP1 and THP1 STING knockout cells in the presence and absence of clonixeril and/or 2',3'-cGAMP. White indicates absence of 2',3'-cGAMP, gray indicates 2',3'-cGAMP is present. Plus (+) indicates addition of clonixeril, minus (-) indicates clonixeril's absence. Gray indicates 2',3'-cGAMP is present. Note the difference in RLU values in the STING -/- experiment.

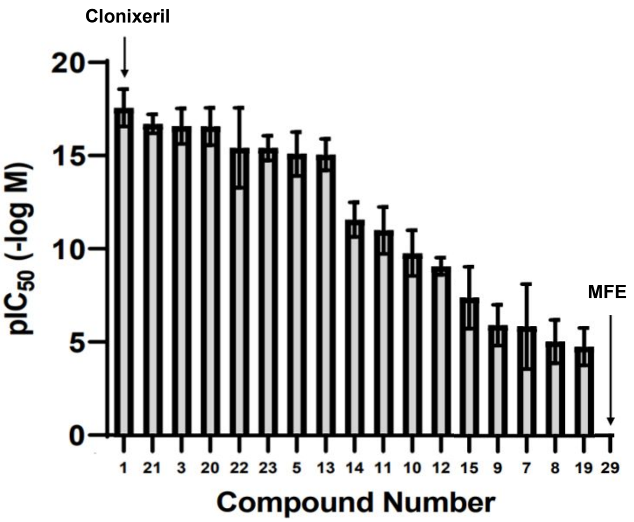

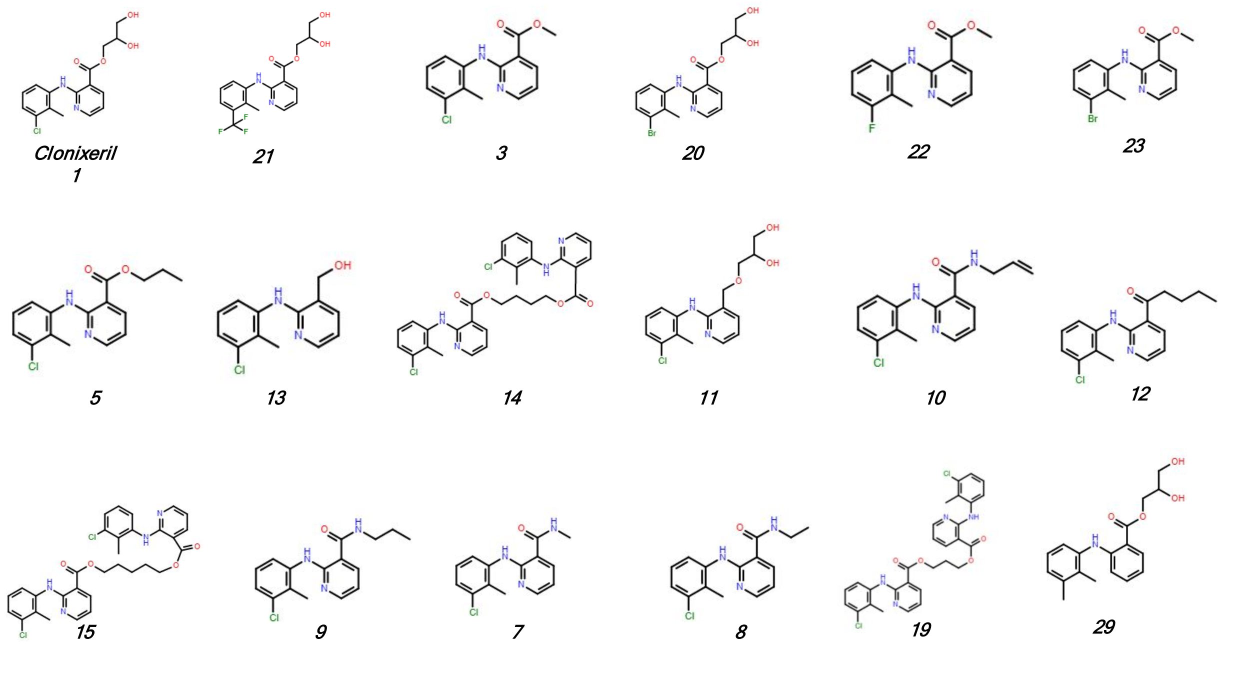

Figure S5. SAR for Clonixeril and Analogs. Summary for 17 selected compounds from an analog library of over 40 compounds screened by MST. IC_{50} values are shown on a log scale. Compound 1 is CXL. Error bars are standard deviations; Chemical Structures of the 17 compounds are shown.

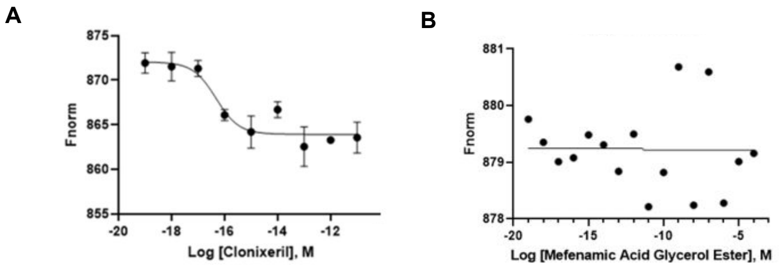

Figure S6. MST for Clonixeril and Mefenamic Acid Glycerol Ester. (A) MST analysis for clonixeril; titration shown is from 100 zM to 10 μM; N=3. (B**)** MST analysis for mefenamic acid glycerol ester; titration shown is from 100 zM to 10 μM; N=3.

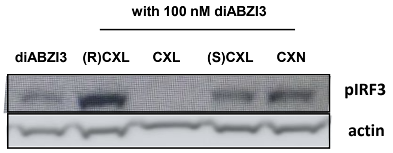

Figure S7. Western Blot for HEK293S cells treated with Clonixeril and its enantiomers. Western blot of HEK293S cells treated with 100 fM clonixeril, R-clonixeril, S-clonixeril, and clonixin in the presence of 100 nM diABZI3.

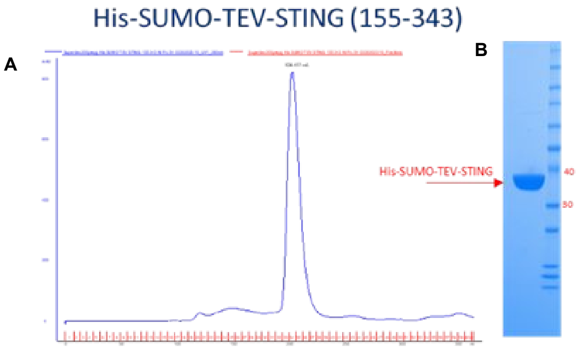

Figure S8. Purity of His-SUMO-TEV-STING. (A) HPLC trace (B) SDS PAGE. Lane 1: Purified His-SUMO-TEV-hSTING CTD; Lane 2 MW marker.

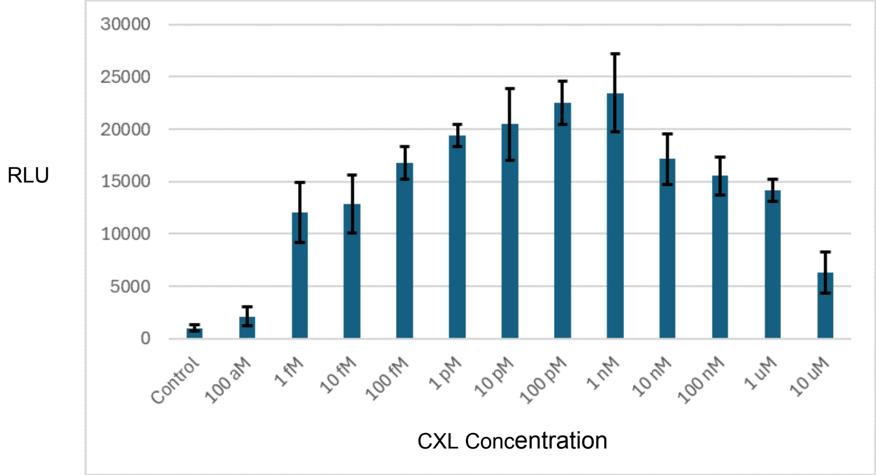

Figure S9. Luciferase Assay Utilizing Monocytic Leukemia (THP-1) cells. Cells (Invivogen THP1 Dual™ KI-hSTING-R232; wild type) were analyzed for activation of the hSTING WT pathway via an IRF3 luciferase reporter. Luminescence is reported in relative luminescence units (RLU). Competition of THP-1 cells treated first with CXL (1hr) and subsequently treated with 2',3'-cGAMP 4μM (9hrs); N=3**.** Full concentration range as compared to **Figure 2B**.
