# Supplementary material for: Discovery of Clonixeril as a Sub-Femtomolar Modulator of the Human STING Receptor
Source: ACS Cent Sci. 2025 Jun 6;11(6):994–1008. doi: 10.1021/acscentsci.4c01982 (PMC12203428; doi:10.1021/acscentsci.4c01982)
Supplement: Supplementary file 2 [file oc4c01982_si_002.pdf]

oc-2024-019822.R1

Name: Peer Review Information for "Discovery of Clonixeril as a Sub-Femtomolar Modulator of the Human STING Receptor"

First Round of Reviewer Comments

Reviewer: 1

Comments to the Author

In this manuscript, Sparks et al, & Guida describe the discovery that clonexeril (CXL), an investigational pro-drug of the NSAID clonexin (CXN) from the 1970s, antagonizes STING receptor activation at astonishingly high potencies, reportedly down into the mid-atomolar (aM) concentration range. Beginning with MD simulations and a small library docking campaign, the authors identify clonexeril as a ~19 µM weak agonist of STING. They subsequently find that at concentrations 10^{11} lower, into the mid-aM range, clonexeril antagonizes STING activiation. The authors support the astounding potency of clonexeril through a series of cell-based and biophysical experiments. These include:

a. DTm measurements via differential scanning fluorimetry, where clonexeril reduced the melting temperature of the STING c-terminal domain (CTD)

b. an IRF3 luminescence reporter assay in THP-1 cells, where clonexeril showed an inverse concentration response, with inhibition increasing as concentration dropped. The maximum inhibition was observed at 100 aM. Realizing that the results were highly unusual, the experiment was repeated in a second laboratory, with different scientists, at UMass Med School.

c. In HEK293 cells, 1 fM clonexeril completely inhibited production of phospho-STING as provoked by the canonical agonist, 2',3'-cGAMP. This experiment was sensitive to the order of addition—if 2',3'-cGAMP was added first, the effect went away. In this experiment, there was no obvious inverse concentration-response until very high concentrations of clonexeril were used (10 µM).

d. Also in HEK293 cells, the authors investigated the effect of IFN-b levels. Again at 1 fM, clonexeril inhibited IFN-b production (about 75% inhibition). Intriguingly here, a normal concentration response was observed, as inhibition was noticeably better at 100 pM and 1 nM than 1 fM (**Figure 3D**).

e. By fluorescence microscopy in HEK293 cells, STING puncta induced by the agonist 2',3'cGAMP were much reduced at concentrations of clonexeril as low as 10 aM.

f. In dynamic light scattering experiments at 50 nM STING CTD protein, 100 aM of racemic clonexeril reduced the size of protein aggregates to about 75 nm, while the *R*-isomer was slightly less potent (judged by aggregate size) and up to 500 fM of the *S*-isomer showed no effect. It seems that the *R*-isomer is the active species.

g. In SPR experiments, no activity could be detected for very low concentrations of clonixeril, which the authors attributed to lack of sensitivity of their chip. At high nM concentrations, binding could be detected.

h. In microscale thermophoresis (MST) experiments, the authors again saw little effect of the compound alone, but saw strong effects, again in the sub-fM concentration range, when competing with the agonist 2',3'-cGAMP.

i. The authors experlore the SAR of clonexeril and find that neither the approved NSAID clonexin—the activated acid form of the pro-drug clonexeril—nor mefenamic acid glycerol ester, which is closely resembles clonexeril (**Figure 1D**)—antagonize STING. They also measure the effects of about 40 other analogs, none of whose structures are shown (**SI Fig S5**). This seems like a gap—the structures should be shown in an ACS journal, I would think. This is especially true since the lack of activity for the highly similar mefenamic acid glycerol ester is disturbing. How sharp is the SAR here?

j. As in the MST experiments, clonexeril's activity at low (fM) concentrations could not be measured. Whereas cGAMP produced a normal looking ITC curve, even at higher concentrations clonexeril the isotherm was weird: it showed no evidence of saturation, but released high amounts of heat in every injection (**Figure 6A-B**, reproduced below):

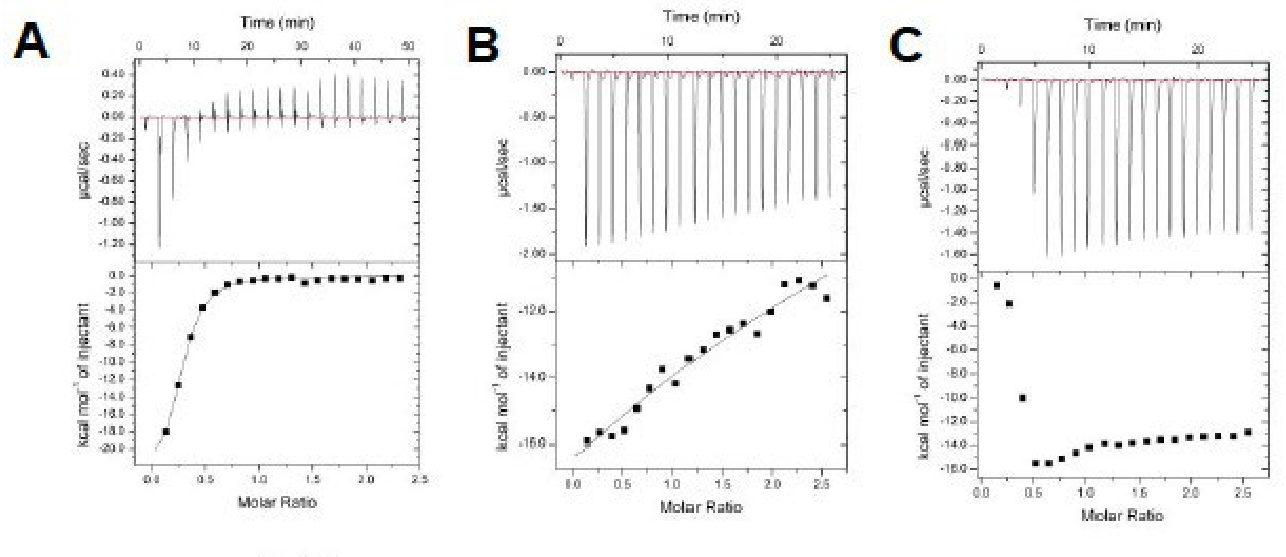

cGAMP clonexeril clonexeril+cGAMP

Accordingly the authors return to what they did in the MST experiment and try to compete cGAMP in the ITC. Here they get what they believe to be a meaningful curve, but one notices that here too the heats evolved are unreasonably high, and don't saturate with injection, even when they are well above a protein:clonexerin molar ratio of 1. So it seems to me to simply reiterate the problem with clonexeril alone.

Taken together, the authors have gone to great lengths to establish what they themselves appear to understand is an observation that will agitate the readership: that a molecule whose direct binding appears to be in the 500 nM to 20 μM range, is having effects at 100 aM concentrations, and that for some of these effects one observed an inverse concentration response. This will feel to some like homeopathy.

I cannot rule out that clonexeril is extremely potent, perhaps via effects on oligomeric states of the receptor, and perhaps even oligomeric states of clonexerin, as are seen in the cryoEM structures of amyloid fibrils bound to PET ligands. It obviously does not have classical dose response, there is something weird going on, and the authors do seem to grasp that. Exactly what that might be they do not make clear, but for a new and wild observation perhaps we cannot expect them to figure everything out. Nevertheless, there are several problems with the manuscript that must be addressed before publication:

Major comments:

1. <u>There is effectively no compound per cell at 100 aM</u>**.** The authors write, "*A simple calculation shows that at 1 fM, the number of molecules per THP-1 cell in our luciferase reporter assay is only approximately 3 molecules per cell.*" The calculation may have been too simple, there is in fact < 0.01 molecules per cell (or, <1 molecule per 100 cells) at 1 fM. At 100 aM, where they typically see the most potent activity, there is <0.001 molecules per cell. At 10 aM, where they moot activity in several of their assays, there is <0.0001 molecules per cell. This can be shown as follows:

Assume a cell radius of 1 µm and a spherical cell, then the cell volume is $4/3pr^3$, or $4/3*p*(1.1*10^{-5})^3$ M^3, or $4/3*p*(10-5)^3$ *1000 L, or: $4.2*10^{-12}$ L.

Multiplying this volume by Avogadro's number, **$2.5*10^{12}$** molecules in the volume of a cell would be 1 M. Thus, the minimum concentration to have at least 1 molecule per cell is 400fM, or $1/2.5*10^{12}$ (others have suggested that the concentration of 1 molecule/cell for a human cell is 100fM (https://bionumbers.hms.harvard.edu/bionumber.aspx?s=n&v=16&id=104519). Taking the lower number of 100 fM, any concentration below this means that they have less than 1 molecule per cell. For 100 aM, they have 1/1000th of a molecule per cell.

Putting this down to cell-cell communication, as they do, strains credulity. The implications here must be reconsidered before the work is suitable for publication. As it is, it will raise a howl among the chattering classes.

2. <u>There is something wrong with the ITC</u>. In both **Fig 6B** and **6C**, far too much heat is released well-past equimolar ratios of conexeril and STING, especially with STING in mid-µM range (and so the ligand too). Basically, every new injection of clonexeril, especially in **6C**, releases another 13 kcal/mol. Ordinarily I'd guess that that owed to the DMSO, but the authors claim they've controlled for that in **6D.** Are they sure about that? I don't see anything in their model that explains this observation.

3. <u>They discuss their SAR, but they don't reveal the vast majority of structures (**Fig S5**)</u>. They can't discuss SAR without showing structures in an ACS journal, IMHO. Moreover, the SAR that they do reveal is disturbing. Why does so small a change in going from clonexeril to mefenamic acid glycerol ester eliminate binding? Revealing more structures (basically, all of them for which they show data), and their effects discussed, would illuminate this area.

4. <u>The inverse concentration-response in the IRF3 luminescence assay is disconcerting</u>. I can understand U-shaped curves, but an actual inverse correlation

between concentration and activity (higher concentration, worse activity) throughout the entire range, from 10^{-17} M and up, is disturbing. And I don't think it's seen in most of their other assays, where a normal concentration response is seen (e.g., in the phospho-STING and in the IFN-b assays in HEK293 cells). How might this be explained?

5. Why does clonexeril reduce temperature of melting in the DSF experiment? By linkage equilibrium, reversible ligand binding should stabilize the protein, otherwise it wouldn't bind. The only exception to this of which I am aware is irreversible ligand binding via covalent modification (which can have the same destabilizing effect as mutating a residue). What do the authors think is going on here?

Minor comments:

6. STING has a shallow binding pocket. Clonexeril is a relatively small molecule with little preorganization. Can the authors reconcile its effects based on complementarity to the receptor? How does the rest of their SAR series fit in with the structural modeling?

7. Along the same lines, while the authors begin the story with MD simulations and docking screens, do they think either is relevant to the discovery of clonexeril, outside of its mid-µM agonist form?

8. Clonixin (CXN) appears to be a STING agonist, and so presumably an activator of the innate immune system. It has a Cmax in humans of about 130 µM, about 10x of its EC_{50} on STING. This would presumably interfere with its activity as an NSAID, or at least be noteworthy over its long use in humans. Any evidence to support this in, for instance, FAERS or in EMRs?

Overall, this is an interesting paper with striking observations of potency, on which the authors have lavished a great deal of work. There could be something fundamental and interesting that the behavior of clonexiril reveals, though what that might be isn't clear from this study. Meanwhile, several of their observations are hard to reconcile with simple considerations of concentration and thermodynamics; these must be figured out before the paper is published.

Author's Response to Peer Review Comments:

Professor Wayne C. Guida
Department of Chemistry
CHE 205
University of South Florida
4202 East Fowler venue
Tampa, Florida 33620

January 20, 2025

Executive Editor, ACS Central Science
RE: Manuscript # oc-2024-019822
“Discovery of Clonixeril as a Sub-Femtomolar Modulator of the Human STING Receptor”.

Dear Editor,

First of all, please thank the referee for us for an excellent review of our manuscript. We’ve attempted to improve it along the lines suggested by the referee, as well as correcting a few errors we noticed when doing so. We believe that we have produced a much better paper in very large part because of the exceptional review. In an attachment, I go through each of the referee’s comments and address them. We hope that in its present form, the manuscript is now acceptable for publication in ACS Central Science. We know that our findings may elicit some controversy, but nothing would please us more than to have other labs involved in reproducing and/or explaining the astonishing cellular potency and inverse dose-response we observe for clonixeril. To date, our laboratory, and two laboratories external to USF, have been able to produce significant data confirming the extreme potency and inverse dose response for CXL in at least three different cell types. In total, five graduate students, three post-docs, and one Ph.D. level senior scientist have been able to reproduce the results described in our manuscript. I am confident that we have observed something fundamental with the results we have obtained. I fully agree with the referee’s comment that we cannot be expected to figure everything out before publication but we believe that our work should be published if for no other reason than it will stimulate the scientific community to provide additional insights.

Sincerely,

Wayne C. Guida, Ph. D.

DEPARTMENT OF CHEMISTRY

University of South Florida | 4202 E Fowler Avenue, | Tampa, FL 33620-4301
813-974-6375 | wguida@usf.edu/

Professor Wayne C. Guida
Department of Chemistry
CHE 205
University of South Florida
4202 East Fowler venue
Tampa, Florida 33620

January 20, 2025

RE: Manuscript # oc-2024-019822
"Discovery of Clonixeril as a Sub-Femtomolar Modulator of the Human STING Receptor".

Response to Reviewer's Comments

Major comments:

*1. There is effectively no compound per cell at 100 aM. The authors write, "A simple calculation shows that at 1 fM, the number of molecules per THP-1 cell in our luciferase reporter assay is only approximately 3 molecules per cell." The calculation may have been too simple, there is in fact < 0.01 molecules per cell (or, <1 molecule per 100 cells) at 1 fM. At 100 aM, where they typically see the most potent activity, there is <0.001 molecules per cell. At 10 aM, where they moot activity in several of their assays, there is <0.0001 molecules per cell. This can be shown as follows: Assume a cell radius of 1 µm and a spherical cell, then the cell volume is 4/3pr3, or 4/3*p*(1.1*10-5)3 M3, or 4/3*p*(10-5)3 *1000 L, or: 4.2*10-12 L. Multiplying this volume by Avogadro's number, 2.5*1012 molecules in the volume of a cell would be 1 M. Thus, the minimum concentration to have at least 1 molecule per cell is 400fM, or 1/2.5*1012 (others have suggested that the concentration of 1 molecule/cell for a human cell is 100fM (https://bionumbers.hms.harvard.edu/bionumber.aspx?s=n&v=16&id=104519). Taking the lower number of 100 fM, any concentration below this means that they have less than 1 molecule per cell. For 100 aM, they have 1/1000th of a molecule per cell. Putting this down to cell-cell communication, as they do, strains credulity. The implications here must be reconsidered before the work is suitable for publication. As it is, it will raise a howl among the chattering classes.*

The point is well taken. The calculation done by the referee is instructive but since we know the approximate cell density in our THP1 assay, we can use that number to more accurately calculate

the number of molecules per cell at 1 fM. Let us assume the range is 2.5x10^5 - 5x10^5 cells per mL. We know the volume of our assay well to be 200 µL, and thus we know how many cells we have in our assay wells (recall that THP-1's are suspension cells and approximately evenly distributed in each well). This allows us to calculate the number of molecules per cell. The calculation (which we now include in the Supplemental Text in our manuscript) is a follows:

DEPARTMENT OF CHEMISTRY

University of South Florida | 4202 E Fowler Avenue, | Tampa, FL 33620-4301
813-974-6375 | wguida@usf.edu/

The volume of the well = 200 µL = 0.2 mL = 2x10^-1mL but cells are typically transferred in 180 µL of media and the volume of the solution is then brought up to 200 µL.

5x10^5 cells/mL x 1.8x10^-1 mL = 9x10^4 cells per assay well

2.5x10^5 cells/mL x 1.8x10^-1 mL= 4.5x10^4 cells per assay well

The concentration of compound at 1 fM = 10^-15 mmol/mL
1x10^-15 mmol/mL * 2x10^-1 mL per well = 2x10-16 mmol
2x10^-16 mmol x 6.02 x 10^20 molecules /mmol = 12.04 x 10^4 molecules per assay well

At a cell density of 2.5x10^5 cells/mL which = 4.5x10^4 cells per assay well, we calculate 2.68 molecules per cell at 1fM.

At a cell density of 5x10^5 cells/mL which = 1.34x10^4 cells per assay well, we calculate 1.34 molecules per cell at 1fM.

Truthfully, we do not have an exact explanation for why we are seeing effects in cells below 1 fM. Given the significant interactions of the STING pathway with the cellular immune response, it is reasonable to expect that transformations in this pathway (both positive and negative) will result in inter-cellular signal transduction. This could occur through secondary messengers (e.g., presence or absence of pIRF3), paracrine signaling of cytokines, or perhaps juxtacrine signaling from cell-to-cell contact. Juxtacrine signaling through gap junctions or other cell-to-cell contact mechanisms could be very intriguing as it is known 2,3-cGAMP travels from cell to cell in this manner (Figure 2 in Decout, *et al. Nat Rev Immunol* **21**, 548–569 (2021). https://doi.org/10.1038/s41577-021-00524-z). It is conceivable that intercellular transport of CXL could increase the probability of its interaction with STING. In other words, perhaps CXL itself does not need to interact with each cell and STING molecule to elicit this overall response, it would only need to interact with a "quorum" of cells that would in turn amplify that information for the local cellular population. Interesting, against this framework, STING itself is known to engage in intercellular trafficking through a mechanism involving autophagy (Gao, *et al.*, *Cell Research* **32**, 1086–1104 (2022); https://doi.org/10.1038/s41422-022-00731-w). We argue that our results should be published despite the above-mentioned uncertainties so that other scientists will be stimulated to help provide answers to the apparent anomaly of the antagonistic behavior of CXL

at concentrations for which there are fractional molecules per cell. We have changed the wording in the revised manuscript as indicated above so that hopefully it will tame howls from the chattering classes. We welcome involvement of other laboratories in finding a solution to this conundrum.

2. There is something wrong with the ITC. In both Fig 6B and 6C, far too much heat is released well-past equimolar ratios of clonexeril and STING, especially with STING in mid-μM range (and so the ligand too). Basically, every new injection of clonexeril, especially in 6C, releases another 13 kcal/mol. Ordinarily I'd guess that that owed to the DMSO, but the authors claim they've controlled for that in 6D. Are they sure about that? I don't see anything in their model that explains this observation.

We are sure we have controlled for DMSO where we had no heat released for the DMSO only control as described in the manuscript. We can provide the raw data in the Supporting Information if recommended by the reviewer. Notably, all ITC experiments were performed multiple times. Furthermore, in numerous experiments cited in our manuscript, we solubilized CXL (without using DMSO) into HBS-P at a starting concentration of 10 micromolar to ensure no DMSO effects were contributing to the astonishing potency we were measuring including SPR, MST, DLS, and in the *in cellulo* pIRF3 WB provided in the Supporting Information (**Figure S7**).

Additionally, because we are convinced that a change in STING-CTD oligomerization is taking place in our ITC experiments, we have found a reference in the literature to ITC experiments that were performed on fibrillation/aggregation of the β-amyloid protein (Ikenoue, *et al.* PNAS **111** (18) 6654-6659 (2014)). In those experiments, a significant amount of heat was liberated as a result of the fibrillation/aggregation event. We now cite that paper in our revised manuscript.

It is worth noting that we do not believe we are dealing with a simple binding event as we show in our proposed mechanism (**Scheme 1** in our manuscript). The very fact that a significant amount of heat is liberated in our ITC experiments indicates, we believe, that oligomers are involved in the mechanism of action and thus we are not dealing with simple 1:1 ligand-protein binding thermodynamics. We believe that what we are seeing in ITC likely involves a simple binding event initially that is followed by a subsequent change in oligomerization state of the protein. We contend that **Figure 6E** fully supports our mechanism regarding the action of CXL at low concentrations. In that figure, a significant amount of heat is released after each injection despite the fact that we are titrating with1.5 fM CXL! We cannot fully explain the amount of heat released as shown in **Figure 6C**, but simply note that the titration is with 400 μM of CXL as opposed to 1.5 fM. We have modified our manuscript to emphasize the point that this experiment actually supports our proposed mechanism (**Scheme 1**). The excerpt from our revised manuscript is show below:

Observation: When we treat HEK293 or THP-1 cells with low concentrations of CXL prior to 2',3'-cGAMP we see potent antagonism. **Explanation:** Here we propose that CXL is binding to apo-STING, which is in its oligomerized autoinhibited state, but at low concentrations an unproductive oligomerization can occur when a single CXL occupied holo-STING molecule interacts with apo-STING non-stoichiometrically (**Scheme 1C**). This becomes a relatively stable inhibited state that cannot be reversed with 2',3'-cGAMP. It is likely that partially closed holo-STING is stabilized by partially closed apo-STING and Ergun, *et al.* provide cryo-EM some evidence for this hypothesis.[6,]

[35] On the other hand, it is likely that there is a profound structural change in the partially occupied apo-oligomers. Our ITC experiments, which granted were done using STING-CTD, support this conjecture because as is shown in **Figure 6E**, every new injection of clonixeril, releases from -7 to -2 kcal/mol of heat over the first 15 injections (out of 19) when 1.5 fM clonixeril is titrated into 40 µM hSTING CTD in the presence of 20 µM 2',3'-cGAMP. We interpret this to mean in the ITC experiment that STING-CTD oligomers preformed with addition of 2',3'-cGAMP are changing oligomerization state in an exothermic manner with clonixeril acting non-stoichiometrically.

3. They discuss their SAR, but they don't reveal the vast majority of structures (Fig S5). They can't discuss SAR without showing structures in an ACS journal, IMHO. Moreover, the SAR that they do reveal is disturbing. Why does so small a change in going from clonexeril to mefenamic acid glycerol ester eliminate binding? Revealing more structures (basically, all of them for which they show data), and their effects discussed, would illuminate this area.

We have now included chemical structures of the analogs in **Figure S5**. We removed some analogs relative to the original manuscript because we cannot show their structure for proprietary reasons. There are examples within the realm of SAR where very simple structural changes can drastically change activity. A good example is the binding of biotin vs. desthiobiotin to streptavidin. The K_d for desthiobiotin is ~4 orders of magnitude weaker than the K_d for biotin. We point out, though, that because we are very likely dealing with complex phenomena, K'_ds measured for small molecules binding to a protein target are not likely to be comparable to the IC_{50}'s or EC_{50}'s we report. In fact, we generally report our "binding" data as EC_{50}'s for this very reason. Accordingly, we don't have a good explanation for mefenamic acid glycerol ester's lack of activity in our MST assay. Moreover, mefenamic acid glycerol ester's lack of activity is too subtle for the current level of computational modeling we have done. As it turns out, it is a good thing to have a so-called decoy analog among our analog set. It would be far more difficult to explain if all of our analogs were as potent as clonixeril. As it is, we generate a reasonable SAR in spite of the fact that we are clearly dealing with very complex thermodynamics that most likely involves a simple binding event initially but is followed by a subsequent change in oligomerization state of the protein.

4. The inverse concentration-response in the IRF3 luminescence assay is disconcerting. I can understand U-shaped curves, but an actual inverse correlation between concentration and activity (higher concentration, worse activity) throughout the entire range, from 10-17 M and up, is disturbing. And I don't think it's seen in most of their other assays, where a normal concentration response is seen (e.g., in the phospho-STING and in the IFN-b assays in HEK293 cells). How might this be explained?

The inverse dose response is more pronounced in our THP-1 cell assay where there is no doubt that there is an inverse dose response. Notably, the trend for inverse dose response was seen in **Figure 3B**, which involves HEK293 cells. We also see it in our native gel (**Figure 6F**) using HEK293T cells transfected with WT STING (referred to as HEK293S cells in our manuscript). These cells have a higher titer of STING than 293's. We are not sure, though, that this is relevant. We did find an example in the literature of an inverse dose-response (Maness, et. al, *Toxicology and Applied Pharmacology* **151**,

135–142 (1998), Figure 2B). Moreover, we do know that that inverted U-shaped curves (or bell-shaped curves) are a somewhat common phenomenon for agonists. Now the "left-hand" side of a bell-shaped curve for an antagonist exhibits an inverse dose-response and we had performed our THP-1 cell assay with 2,3-cGAMP as the activator examining a wider concentration range of CXL than is shown in **Figure 2B**. We now show the graph with the full concentration range of CXL in **Figure S9** which is very clearly bell-shaped. We prefer to only show the low concentration range in the main body of the manuscript to emphasize the inverse dose-response relationship and for comparison with **Figure 2C** where we have not examined higher concentrations of CXL with DiABZI3 as the activator. We do realize that an inverse dose response for an antagonist is a "two-edge sword" for molecules as potent as CXL. This is precisely because of the issue of the number of molecules per cell which can make it difficult to obtain accurate EC_{50}'s at very low concentrations.

5. Why does clonexeril reduce temperature of melting in the DSF experiment? By linkage equilibrium, reversible ligand binding should stabilize the protein, otherwise it wouldn't bind. The only exception to this of which I am aware is irreversible ligand binding via covalent modification (which can have the same destabilizing effect as mutating a residue). What do the authors think is going on here?

We suspect that once again, we are not dealing with a 1:1 binding event or even a single event. We are convinced that oligomerization is in play when CXL engages STING as is true for 2',3'-cGAMP. This experiment, like all of our other biochemical/biophysical experiments, was done with the C-terminal domain of STING. In this experiment, CTD STING is likely already oligomerized to some extent. Later we learned, using DLS, that even one freeze-thaw cycle can induce the STING CTD to oligomerize. Apparently, the addition of CXL changes the oligomerization state in such a way that the melting temperature is reduced. We simply don't have enough information to form a reasonable hypothesis about what is happening in this experiment from a structural point of view.

Minor comments:

6. STING has a shallow binding pocket. Clonexeril is a relatively small molecule with little pre-organization. Can the authors reconcile its effects based on complementarity to the receptor? How does the rest of their SAR series fit in with the structural modeling?

Yes. The binding pocket is somewhat shallow, but it is large enough to accommodate a cyclic-dinucleotide. In fact, a main question we pondered was whether one or two molecules of CXL were binding to the pocket. We describe in the Supporting Information our computational procedure (Site-Restriction Virtual Screening) to determine the likelihood of two molecules binding to the pocket. We predicted that it would be two, as we mention in the manuscript. We now show that a dimer of the carboxylic acid, clonixin,

linked as a diester with 1,4-butane-diol (Compound 14 in **Figure S5**) is quite active in our MST assay, whereas the propane-diol linked dimer and pentane-diol linked dimers were much less active. Once again, we speculate, that binding of the ligand to the pocket is linked to a subsequent STING oligomerization or de-oligomerization event. More details about the SAR of our analogs are to be included in a *J. Med. Chem.* paper in preparation.

7. Along the same lines, while the authors begin the story with MD simulations and docking screens, do they think either is relevant to the discovery of clonexeril, outside of its mid-μM agonist form?

Discovery, probably not. But note our answer to minor comment 6. Initially we were searching for agonists. But the fact that CXL is a weak agonist, is what led us to question whether it might be a partial agonist, thus exhibiting antagonistic activity as well. We were, in fact, totally surprised by its potency as an antagonist.

8. Clonixin (CXN) appears to be a STING agonist, and so presumably an activator of the innate immune system. It has a Cmax in humans of about 130 μM, about 10x of its EC50 on STING. This would presumably interfere with its activity as an NSAID, or at least be noteworthy over its long use in humans. Any evidence to support this in, for instance, FAERS or in EMRs?

This is an interesting question but it is a bit beyond the scope of this study. That being said, we have no strong evidence that CXN is an agonist, but see **Figure S7**, where it seems to enhance the potency of diABZI3, a known agonist. Certainly CXL is an agonist at high concentrations (EC_{50} = 20 μM; **Figure 2A**). We suspect that CXN is as well, given its EC_{50} of 637 nM by SPR (**Table S1**). We are conducting experiments involving our THP-1 cell assay to determine whether CXN is an agonist *in cellulo* but do not have publishable results yet. Nonetheless, the reviewer's point is very well taken because the hydrolysis product of CXL is CXN. We show in the Supporting Information (**Figure S4B**) that CXL's hydrolysis is surprisingly slow at pH=2.0. Nevertheless, *in vivo*, in the presence of esterases, CXN may be produced rapidly. As it turns out, we have another project that involves data mining using the FAERS database (we don't have access to patient data in EMR). Below is what we have discovered about CXN. We do suggest that this not be added to our manuscript. We will be submitting a manuscript to *J. Med. Chem.* in due course that describes all of our analogs of CXN and goes into more detail about the therapeutic utility of CXL and its analogs. We also point out that to really answer the question we would need a control dataset with matched demographics and we'd have to define what would constitute interference with NSAID activity. Pain is not informative. We expect pain also comes up as an adverse event for other NSAIDs.

Preliminary Data from FAERS:

We ran Clonixin through FAERS and pulled up indications and statistics corresponding to adverse events reported through FAERs. We only looked at indications where more than 1 report was in

FAERs database. For this study, we did not run another NSAID as a control to compare adverse events when probing our database.

Adverse Event	Reports	Frequency	PRR	ROR	CI (Lower 95%)	CI (Upper 95%)	CI < 1
pain	21	0.1909090909	1.52386959	1.534498828	0.9961659347	2.363749423	FALSE
headache	14	0.1272727273	1.442675517	1.448623173	0.8549177556	2.454632721	FALSE
back pain	10	0.09090909091	2.760626344	2.777458335	1.489915507	5.177659249	FALSE
musculoskeletal stiffness	9	0.08181818182	5.273312147	5.310045489	2.754828103	10.23533304	FALSE
bone pain	9	0.08181818182	10.61272486	10.69535573	5.548086312	20.61803437	FALSE
migraine	7	0.06363636364	4.269509248	4.291326754	2.040544916	9.02478802	FALSE
spinal pain	5	0.04545454545	20.0771857	20.16794301	8.372153081	48.58319254	FALSE
inflammation	2	0.01818181818	1.926835096	1.928593797	0.4816498116	7.722361647	FALSE

FAERS classifies severity of an adverse event as follows: Grade 1: Mild, Grade 2: Moderate, Grade 3: Severe, Grade 4: Life threatening, and Grade 5: Death. For purposes of our tool we used rubric of 0.00 < ROR < 1.68: Safe, 1.68 < ROR < 3.47 and If lower bound of the CI > 1: Likely unsafe, 3.47 < ROR < 6.71 and If lower bound of the CI > 1: Unsafe ROR >. 6.71 and if lower bound of the CI > 1: Highly unsafe. The data suggest CXN use in humans could present issues with its function as an NSAID for common indications such as back pain, musculoskeletal stiffness, bone pain, migraine, and spinal pain. Note: PRR = Proportional Reporting Ratio; ROR = Relative Odds Ratio; CI = Confidence Interval.

Response to Referee's Initial and Final Narrative with embedded questions:

c. In HEK293 cells, 1 fM clonexeril completely inhibited production of phosphoSTING as provoked by the canonical agonist, 2',3'-cGAMP. This experiment was sensitive to the order of addition—if 2',3'-cGAMP was added first, the effect went away. In this experiment, there was no obvious inverse concentration-response until very high concentrations of clonexeril were used (10 µM).

We agree that the inverse dose response becomes obvious at 10 *µM* and discuss this in more detail below in response to the Major comments.

f. In dynamic light scattering experiments at 50 nM STING CTD protein, 100 aM of racemic clonexeril reduced the size of protein aggregates to about 75 nm, while the R-isomer was slightly less potent (judged by aggregate size) and up to 500 fM of the S-isomer showed noeffect. It seems that the R-isomer is the active species.

Yes. It seems that way by DLS and MST. Cellular studies are in progress and eventually will be reported in detail in a *J. Med. Chem.* manuscript in preparation. Preliminary results are already included in our current manuscript which suggest that the R-enantiomer is an agonist (**Figure S7**).

h. In microscale thermophoresis (MST) experiments, the authors again saw little effect of the compound alone, but saw strong effects, again in the sub-fM concentration range, when competing with the agonist 2',3'-cGAMP.

Yes, this is also intriguing. Current MST instruments have a picomolar detection limit. We discovered that by doing the experiment *via* competition we could use MST to detect effects at the attomolar level. We believe it is important to describe our methodology in greater detail than possible in the present manuscript. We have a manuscript under revision for ACS Measurement Science that does just that. This takes advantage of the nanomolar binding affinity of 2',3'-cGAMP to allow measurement beyond the detection limit of current MST technology.

i. The authors experlore the SAR of clonexeril and find that neither the approved NSAID clonexin—the activated acid form of the pro-drug clonexeril—nor mefenamic acid glycerol ester, which is closely resembles clonexeril (Figure 1D)—antagonize STING. They also measure the effects of about 40 other analogs, none of whose structures are shown (SI Fig S5). This seems like a gap—the structures should be shown in an ACS journal, I would think. This is especially true since the lack of activity for the highly similar mefenamic acid glycerol ester is disturbing. How sharp is the SAR here?

We addressed this in our response to the major comments but we would add that the SAR is pretty sharp. We would caution that MST is not a highly precise instrument. That being said, we confirmed our MST with clonixeril *via* ITC, DLS, and mass photometry. It is possible that MFE does have weak activity.

Epilog

Overall, this is an interesting paper with striking observations of potency, on which the authors have lavished a great deal of work. There could be something fundamental and interesting that the behavior of clonexiril reveals, though what that might be isn't clear from this study. Meanwhile, several of their observations are hard to reconcile

with simple considerations of concentration and thermodynamics; these must be figured out before the paper is published.

We believe that there is something fundamental going on that CXL's interaction with STING is revealing to us. Although there are pieces of the puzzle that will most likely have to await planned CryoEM studies, we reiterate that we believe we are not dealing with simple equilibrium thermodynamics. We submit that CXL is acting in a non-stoichiometric fashion which we discuss relative to our proposed mechanism (**Scheme 1**). As previously stated, we have modified the narrative about our mechanism to clarify how it is consistent with the results of our ITC experiment shown in **Figure 6E**. Like all proposed mechanisms, ours is subject to revision as additional experiments are done by us and perhaps others. We look forward to the day when things are crystal clear.
